# The RpoS Gatekeeper in *Borrelia burgdorferi*: An Invariant Regulatory Scheme That Promotes Spirochete Persistence in Reservoir Hosts and Niche Diversity

**DOI:** 10.3389/fmicb.2019.01923

**Published:** 2019-08-21

**Authors:** Melissa J. Caimano, Ashley M. Groshong, Alexia Belperron, Jialing Mao, Kelly L. Hawley, Amit Luthra, Danielle E. Graham, Christopher G. Earnhart, Richard T. Marconi, Linda K. Bockenstedt, Jon S. Blevins, Justin D. Radolf

**Affiliations:** ^1^Department of Medicine, UConn Health, Farmington, CT, United States; ^2^Department of Pediatrics, UConn Health, Farmington, CT, United States; ^3^Department of Molecular Biology and Biophysics, UConn Health, Farmington, CT, United States; ^4^Department of Internal Medicine, Section of Rheumatology, Allergy and Immunology, Yale School of Medicine, Yale University, New Haven, CT, United States; ^5^Division of Infectious Diseases and Immunology, Connecticut Children’s Medical Center, Hartford, CT, United States; ^6^Department of Microbiology and Immunology, University of Arkansas for Medical Sciences, Little Rock, AR, United States; ^7^Department of Microbiology and Immunology, Virginia Commonwealth University Medical Center, Richmond, VA, United States; ^8^Department of Genetics and Genome Science, UConn Health, Farmington, CT, United States; ^9^Department of Immunology, UConn Health, Farmington, CT, United States

**Keywords:** *Borrelia burgdorferi*, Lyme disease, rpoS, persistence, gene regulation, vector borne disease, sigma factor, host adaptation

## Abstract

Maintenance of *Borrelia burgdorferi* within its enzootic cycle requires a complex regulatory pathway involving the alternative σ factors RpoN and RpoS and two ancillary *trans*-acting factors, BosR and Rrp2. Activation of this pathway occurs within ticks during the nymphal blood meal when RpoS, the effector σ factor, transcribes genes required for tick transmission and mammalian infection. RpoS also exerts a ‘gatekeeper’ function by repressing σ^70^-dependent tick phase genes (e.g., *ospA*, *lp6.6*). Herein, we undertook a broad examination of RpoS functionality throughout the enzootic cycle, beginning with modeling to confirm that this alternative σ factor is a ‘genuine’ RpoS homolog. Using a novel dual color reporter system, we established at the single spirochete level that *ospA* is expressed in nymphal midguts throughout transmission and is not downregulated until spirochetes have been transmitted to a naïve host. Although it is well established that *rpoS*/RpoS is expressed throughout infection, its requirement for persistent infection has not been demonstrated. Plasmid retention studies using a *trans*-complemented Δ*rpoS* mutant demonstrated that (i) RpoS is required for maximal fitness throughout the mammalian phase and (ii) RpoS represses tick phase genes until spirochetes are acquired by a naïve vector. By transposon mutant screening, we established that *bba34/oppA5*, the only OppA oligopeptide-binding protein controlled by RpoS, is a *bona fide* persistence gene. Lastly, comparison of the strain 297 and B31 RpoS DMC regulons identified two cohorts of RpoS-regulated genes. The first consists of highly conserved syntenic genes that are similarly regulated by RpoS in both strains and likely required for maintenance of *B. burgdorferi* sensu stricto strains in the wild. The second includes RpoS-regulated plasmid-encoded variable surface lipoproteins *ospC*, *dbpA* and members of the *ospE/ospF/elp*, *mlp*, *revA*, and Pfam54 paralogous gene families, all of which have evolved *via* inter- and intra-strain recombination. Thus, while the RpoN/RpoS pathway regulates a ‘core’ group of orthologous genes, diversity within RpoS regulons of different strains could be an important determinant of reservoir host range as well as spirochete virulence.

## Introduction

Lyme disease, caused by the spirochete *Borrelia burgdorferi*, is the predominant arthropod-borne infection in the United States (Schwartz et al., 2017). Maintenance of the spirochete’s dual-host lifecycle requires migration between an arthropod vector, *Ixodes scapularis*, and a reservoir host, typically small rodents (Radolf et al., 2012; Steere et al., 2016; Stanek and Strle, 2018). Immature *I. scapularis* ticks are able to feed on a wide range of mammalian and avian hosts, leading to geographic expansion of the spirochete’s enzootic cycle (Keirans et al., 1996); the generalist feeding behavior of *I. scapularis* also is responsible for the incidental infection of humans (Piesman and Schwan, 2010). When naïve larvae feed on an infected host, spirochetes acquired with the blood meal colonize the tick midgut and, following a burst of replication, enter a quiescent state post-molt (Burgdorfer et al., 1982; Piesman and Schwan, 2010; Stewart and Rosa, 2018). During transmission, the nymphal blood meal provides the diverse nutrients required by spirochetes to replicate exponentially along with the environmental signals that activate the genetic programs responsible for transmission and early infection (Radolf et al., 2012; Caimano et al., 2016; Stewart and Rosa, 2018). Environmental priming of *B. burgdorferi* by the blood meal also dramatically reduces the numbers of organisms required to establish infection *via* the arthropod route compared to needle-inoculation (Kasumba et al., 2016). Once in the dermis, spirochetes must adapt to changes in nutrient availability, evade local host defenses, and sense environmental cues for dissemination, a process referred to as mammalian host-adaptation (Radolf et al., 2012; Caimano et al., 2016; Stewart and Rosa, 2018). In order to be sustained in nature, following dissemination, spirochetes must persist within the dermis of an infected reservoir host at sufficiently high numbers to be re-acquired by naïve ticks, typically larvae (Ribeiro et al., 1987; Stewart and Rosa, 2018).

Adaptation of spirochetes to the arthropod or mammalian host milieu is exquisitely regulated, with acquisition, transmission and infection giving rise to distinct transcriptional profiles (Iyer et al., 2015). Differential gene expression in *B. burgdorferi* is coordinated, in large part, by two global regulatory networks – the Hk1/Rrp1 two component system (Rogers et al., 2009; Caimano et al., 2011, 2015; He et al., 2011; Kostick et al., 2011) and the BosR/Rrp2/RpoN/RpoS pathway (Hubner et al., 2001; Boylan et al., 2003; Yang et al., 2003a; Hyde et al., 2009; Ouyang et al., 2009, 2011; Groshong et al., 2012) (hereafter referred to as the RpoN/RpoS pathway). These two pathways serve distinct functions during the enzootic cycle. The Hk1/Rrp1 two component system (TCS) is active during the larval and nymphal blood meals and exerts its regulatory effect *via* synthesis of the bacterial second messenger c-di-GMP (Rogers et al., 2009; Caimano et al., 2011, 2015; He et al., 2011; Kostick et al., 2011). Spirochetes lacking either Hk1 or Rrp1 are unable to survive within fed midguts during acquisition or transmission (Caimano et al., 2011, 2015; He et al., 2011). The RpoN/RpoS pathway, in contrast, is active in ticks only during transmission when RpoS, the primary effector (Caimano et al., 2007; Samuels, 2011; Ouyang et al., 2012), upregulates the expression of genes required for tick-to-mammal transmission (Gilmore et al., 2010; Eggers et al., 2011; Patton et al., 2011, 2013; Dunham-Ems et al., 2012) and the establishment of infection in mammals (Grimm et al., 2004; Tilly et al., 2006; Blevins et al., 2008; Shi et al., 2008; Dunham-Ems et al., 2012). RpoS-deficient organisms are avirulent by both tick- and needle-inoculation (Caimano et al., 2004; Dunham-Ems et al., 2012). While *rpoS* and at least some RpoS-upregulated genes are transcribed throughout the mammalian host phase (Liang et al., 2002b; Gilmore et al., 2008, 2007; Ouyang et al., 2012), the contribution(s) of RpoS-dependent gene products to persistence has yet to be determined. RpoS not only upregulates genes required for transmission and infectivity but it also exerts a ‘gatekeeper’ function in mammals by repressing σ^70^-dependent tick phase genes (Caimano et al., 2005, 2007). These RpoS-repressed genes include *bba62/lp6.6*, encoding a ∼6-kDa subsurface lipoprotein capable of forming outer membrane-associated multiprotein complexes (Lahdenne et al., 1997; Promnares et al., 2009), the *glp* operon (*bb0240-0243*) required for uptake and utilization of glycerol (He et al., 2011; Pappas et al., 2011; Caimano et al., 2015), and *ospA*, which promotes midgut colonization within the vector (Caimano et al., 2005, 2007; Pappas et al., 2011; Iyer et al., 2015; Grove et al., 2017). Importantly, there are divergent viewpoints as to when RpoS-mediated repression occurs during the enzootic cycle. Several studies, including a seminal report by Schwan et al. (1995), suggest that downregulation of *ospA* occurs in parallel with transcription of *rpoS* and consequent upregulation of *ospC* during transmission (Barthold et al., 1995; Schwan et al., 1995; de Silva et al., 1996; Schwan and Piesman, 2000). Pal et al. (2000, 2001, 2004) postulated that downregulation of OspA is required for spirochetes to detach from the midgut epithelium. On the other hand, there now exists a substantial body of evidence arguing that spirochetes within feeding nymphs express *ospA* transcript and protein throughout transmission (Schwan et al., 1995; Fingerle et al., 1998; Schwan and Piesman, 2000; Belperron and Bockenstedt, 2001; Ohnishi et al., 2001; Mulay et al., 2009; Promnares et al., 2009; Dunham-Ems et al., 2012; Ouyang et al., 2012; Iyer et al., 2015). Consistent with these studies, we demonstrated by intravital imaging that spirochetes remain adherent to the midgut epithelium surface throughout most of the nymphal blood meal, detaching only after the epithelium has undergone extensive remodeling (Dunham-Ems et al., 2009, 2012). Battisti et al. (2008) proposed that OspA serves a protective function that is required throughout the blood meal. Clarification of the time frame for *ospA* downregulation has important implications for understanding OspA function as well as the mechanism(s) whereby this and other tick phase genes are repressed by RpoS.

In this study, we undertook a broad examination of the RpoN/RpoS pathway in order to address fundamental questions regarding the nature of RpoS, its gatekeeper function and the genes subject to its control. By homology modeling, we found that the alternative σ factor of *B. burgdorferi* annotated as RpoS (BB0771) (Fraser et al., 1997) contains all of the structural hallmarks of a *bona fide* RpoS despite its considerable phylogenetic divergence from Gram-negative RpoS homologs (Chiang and Schellhorn, 2010; Hengge, 2011). Using transcriptional reporters and mutagenesis in concert with our dialysis membrane chamber (DMC) peritoneal cultivation system (Akins et al., 1998; Caimano, 2018), we demonstrated that RpoS-mediated repression of *ospA* is a mammalian host phenomenon requiring an intact RpoN/RpoS pathway. We also provide evidence that RpoS-upregulated gene products are required beyond the acute stage of infection in mice, during which the RpoS gatekeeper function represses tick-phase genes until spirochetes are acquired by a naïve vector. By transposon mutant screening, we establish *bba34/oppA5*, the only RpoS-regulated OppA oligopeptide-binding protein (Medrano et al., 2007; Groshong et al., 2017), as a prototypical persistence gene. Lastly, by comparative transcriptomics of strains B31 and 297, we identified two broad categories of RpoS-regulated genes. The first consists of highly conserved syntenic (i.e., orthologous) genes that are similarly regulated in both strains and likely required for maintenance of *B. burgdorferi* in nature. The second includes plasmid-encoded variable surface lipoproteins *ospC*, *dbpA* and members of the *ospE/ospF/elp*, *mlp*, *revA*, and Pfam54 paralogous gene families, all of which have evolved *via* inter- and intra-strain recombination. Thus, while the RpoN/RpoS pathway regulates a ‘core’ group of orthologous genes with conserved functions, diversity within RpoS regulons of different strains could be an important determinant of reservoir host range as well as the degree of virulence for humans. Our results illustrate the potential utility of transcriptomics as a platform for relating the evolutionary genetics of *B. burgdorferi* to the geographic expansion of Lyme disease.

## Materials and Methods

### Ethics Statement

This study was carried out in accordance with protocols reviewed and approved by Institutional Animal Care and Use Committees from the UConn Health [Animal Welfare Assurance (AWA) number A347-01], Yale University (AWA number D16-04116) and University of Arkansas for Medical Sciences (AWA number A3063-01) following recommendations in the *Guide for the Care and Use of Laboratory Animals* of the National Institutes of Health (National Research Council, 2011).

### Culture and Maintenance of Bacterial Strains

*Escherichia coli* strains were maintained in Lysogeny broth (LB) or LB agar; when appropriate, antibiotic(s) (kanamycin, 50 μg/ml; ampicillin, 100 μg/ml; spectinomycin, 100 μg/ml; and/or gentamicin, 5 μg/ml) were added. *B. burgdorferi* isolates were cultivated in modified BSK-II (Pollack et al., 1993) supplemented with 6% rabbit serum (Pel-Freeze Biologicals, Rogers, AR, United States); when appropriate, antibiotics (kanamycin, 400 μg/ml; streptomycin, 100 μg/ml; gentamicin, 50 μg/ml) were added. *B. burgdorferi* temperature-shift experiments were performed as previously described (Caimano et al., 2004). The plasmid content of *B. burgdorferi* strain 297 and B31 isolates was monitored as previously described (Eggers et al., 2002; Elias et al., 2002). *Borrelia* cultures were passaged no more than three times *in vitro* prior to use in experiments. Detailed descriptions of the *B. burgdorferi* strains used in these studies are presented in Supplementary Table 1.

### DNA Manipulations and Routine Cloning

Routine molecular cloning and plasmid propagation were performed using *E. coli* Top10 cells (Life Technologies, Grand Island, NY, United States). Plasmid DNAs were purified using Qiagen Prep kits (Qiagen, Valencia, CA, United States). Routine and high-fidelity PCR amplifications were performed using Choice *Taq* (Denville Scientific, Metuchen, NJ, United States) and CloneAmp HiFi (TaKaRa Bio USA, Mountain View, CA, United States), respectively. Oligonucleotide primers used in these studies (Supplementary Table 2) were purchased from Sigma-Aldrich (St. Louis, MO, United States). Nucleotide sequencing was performed by Genewiz, Inc. (Cambridge, MA, United States).

### SDS–PAGE and Immunoblot Analyses

Whole-cell lysates prepared from spirochetes cultivated to late logarithmic phase at 37°C following temperature-shift *in vitro* (∼7 × 10^7^ spirochetes per ml) were separated on 12.5% separating polyacrylamide mini-gels and visualized by silver staining as previously described (Caimano et al., 2007). Rabbit polyclonal antiserum against RpoS (Seshu et al., 2004) was generously provided by Jon Skare (Texas A&M University). Polyclonal antisera against strain B31 OspC and OspA used in these studies were generated by hyper-immunizing female Sprague-Dawley rats (150 to 174 g) with the corresponding purified full-length recombinant His-tagged protein lacking the N-terminal signal sequence as previously described (Akins et al., 1998). Whole cell lysate or purified recombinant proteins were transferred to reinforced nitrocellulose (GE Healthcare Life Sciences, Pittsburgh, PA, United States) and incubated overnight with rat polyclonal antiserum against RpoS (Seshu et al., 2004), FlaB (Caimano et al., 2005), OspC, DbpA (Hagman et al., 1998), or OspA, each diluted 1:1000 – 1:3000, followed by the goat horseradish peroxidase-conjugated secondary antibody (Southern Biotechnology Associates, Birmingham, AL, United States) diluted 1:30,000 – 1:50,000. Immunoblots were developed using the SuperSignal West Pico chemiluminescence substrate (Pierce, Rockford, IL, United States).

### Routine Infection Studies

Four- to eight-week-old, female C3H/HeJ (Jackson Laboratories, Bar Harbor, ME, United States) or C3H/HeNHsd (Envigo RMS Inc., Indianapolis, IN, United States) mice were inoculated with 10^4^ or 10^5^ organisms *via* intradermal/subcutaneous injection. Transmission by infected nymphs was assessed using ∼15 nymphs per mouse confined to a capsule affixed to the backs of naïve C3H/HeJ mice as previously described (Mulay et al., 2009). Routine serology was performed using blood collected from mice 2 and/or 4 weeks following infection by needle- or tick-inoculation. Tissues (ear, skin, tibiotarsal joints, heart, and/or bladder) were harvested 4 to 9 weeks post-infection for culturing in BSK-II. Cultures were examined weekly for the presence of spirochetes by dark-field microscopy for up to 8 weeks.

### Generation of Dual Color P*ospA-gfp/* P*flgB-tdTomato B. burgdorferi*

A suicide vector (pMC3752) containing green fluorescent protein (*gfp*) under the control of the *ospA* promoter (P*ospA*) was generated using pBRV2 as the starting vector. Briefly, pBRV2 was generated by TA-cloning an amplicon containing *bbb18-bbb22* (nucleotides 16142-18693) from *B. burgdorferi strain* B31 cp26 (Accession number AE000792.1) into pCR2.1 (Invitrogen, Carlsbad, CA, United States); this region, which includes *ospC* (*bbb19*), is used for insertion into the endogenous cp26 plasmid by allelic exchange. Promoter-less GFP, P*flgB-aadA (*conferring resistance to streptomycin in *B. burgdorferi)* (Frank et al., 2003) and constitutive *tdTomato* genes were cloned into the *bbb21-22* intergenic region. Transcription of *tdTomato* is driven by readthrough from *aadA*. The *ospC* ribosomal binding site also was included upstream of *tdTomato* to enhance translation. The P*ospA* promoter was inserted directly upstream of *gfp* using the Clontech In-Fusion^®^ HD EcoDry^TM^ Cloning Plus system (TaKaRa Bio, United States). Briefly, a P*ospA* fragment containing 169 bp of sequence upstream of the *ospA* translational start site (Grove et al., 2017) was amplified from B31 5A18 NP1 (Kawabata et al., 2004) genomic DNA using CloneAmp HiFi premix with OspA-BRV2-F/OspA-BRV2-R, each containing ∼15 bp of overlap with the *Nhe*I site in pBRV2. ∼100 ng each of gel-purified P*ospA* amplicon and *Nhe*I-digested vector were combined by In-fusion EcoDry cloning according to the manufacturer’s instructions and transformants selected on LB plates containing kanamycin; one positive *E. coli* clone, MC3752, was selected. The sequence for pMC3752 has been deposited in GenBank (Accession number MN186288). Purified pMC3752 (∼25 μg) was electroporated into B31 5A18 NP1 (Kawabata et al., 2004) as previously described (Samuels, 1995; Samuels et al., 2018). Transformants were selected in BSK-II containing appropriate antibiotics in 96-well microtiter plates and screened for GFP by epifluorescence microscopy and PCR using primers PlessSS-F/PlessSS-R within the streptomycin-resistance cassette. One positive clone, BbP1981, containing a full complement of endogenous plasmids was selected.

### Flow Cytometry

Aliquots containing ∼5 × 10^7^ spirochetes grown at 37°C *in vitro* following temperature-shift were pelleted at 8,000 × *g* for 5 min and the resulting cell pellets were washed once with 1 ml of TN buffer (10 mM Tris–HCl, pH 8.0, 1 mM EDTA, 100 mM NaCl). Washed pellets were resuspended in 0.5 mls of FACSFlow^TM^ buffer (BD Biosciences, San Jose, CA, United States) and mixed end-over-end to ensure an even cell suspension. After 10 min, 0.5 mls of FACSFlow^TM^ buffer containing 2% paraformaldehyde and 10 μg/ml 4′,6-diamidino-2-phenylindole (DAPI) was added and cells were fixed for 10–15 min at room temperature. Cells were then washed once with FACSFlow^TM^ buffer and resuspended with 500 μl of FACSFlow^TM^ buffer. A minimum of 10,000 events were acquired using a BD LSR-II flow cytometer and FACSDIVA^TM^ software (BD Biosciences). Analysis was performed using FlowJo V10.4.1 for Mac (FlowJo LLC, Ashland, OR, United States). The mean fluorescence intensity (MFI) of GFP were determined for DAPI-positive spirochetes for each strain. MFI values for each strain were compared in GraphPad Prism (version 7) using a Mann–Whitney test with *p* ≤ 0.05 considered significant.

### Imaging Fluorescent *B. burgdorferi* Within Infected Nymphs During Acquisition and Transmission

Acquisition studies were performed using naïve pathogen-free *Ixodes scapularis* larvae or nymphs fed on infected C3H/HeJ or *Myd88-/-* mice as previously described (Mulay et al., 2009; Caimano et al., 2015). Transmission studies were performed as described previously (Mulay et al., 2009). To monitor expression of P*ospA-gfp* over the course of tick feeding, partially to fully fed nymphs were forcibly removed from mice beginning ∼24 hrs post-placement. Midguts were dissected into PBS, mounted in PBS or VectaShield, and visualized by epifluorescence microscopy on an Olympus BX41 microscope equipped with a Retiga Exi (QImaging, Surrey, BC, Canada) camera. Images were acquired using a 40× (1.4 NA) oil immersion objective with QCapture software v. 2.1 (QImaging).

### Imaging of *B. burgdorferi* Within Murine Tissues by Multiphoton Confocal Microscopy

*Myd88*^–/–^ mice were inoculated with 10^5^ organisms in 20 μl BSK-II at the base of either ear and then imaged at 24, 48, 72, and 96 h post-inoculation (inoculation site) or 7, 8, 13, 30, and 110 days post-inoculation (inoculation site or distal ear). Two-photon imaging of ears and patellofemoral joint and surrounding tendons was performed as previously described (Bockenstedt et al., 2014; Pineda et al., 2015; Belperron et al., 2018). Images were acquired using an Olympus BX61WI fluorescence microscope with a ×20, 0.95 NA water immersion Olympus objective and dedicated single-beam LaVision TriM scanning laser (LaVision Biotec) that was controlled by Imspector software. The microscope was outfitted with a Chameleon Vision II Ti:Sapphire Laser (Coherent) with pulse precompensation. Emission wavelengths of 390–480 nm (blue, DAPI), 500–550 nm (green, GFP), and 565–665 nm (orange-red, tdTomato) were collected with an array of three photomultiplier tubes (Hamamatsu). Images were acquired at a ≅940 nm wavelength. The imaging depth and laser power (12.5–40% for ears and 20–25% for joints) varied depending on the tissue thickness and fluorescence intensity of the spirochetes. Second harmonic generation (SHG) of collagen fibers allowed visualization of the murine tissue without the need for fluorescent labeling. When obtaining Z-stacks (1–3 μm per step), the laser power was continuously adjusted linearly upward as the depth of scanning increased. Scanfield dimensions were between 300 and 500 μm^2^, pixel dimensions were 500 × 500. A minimum of 20 fields of view per sample were examined. Imaris software (version 9) was used for all image analyses.

### Cryosectioning and Imaging of Embedded Tissues

Ear and patellofemoral joint tissues were fixed in 4% paraformaldehyde overnight at 4°C, rinsed extensively with PBS, incubated in ≥10 volumes of 30% sucrose for at least 1 h, and then embedded in OCT using a 2-methyl-butane/dry ice/ethanol bath. Seven μm sections were cut using a Leica CM3050 S cryostat. Unstained cryosections were mounted in Flouromount-G (Southern Biotech) and imaged using an Olympus BX-40 fluorescent microscope with UPLanF1, 40×/0.17 and UPLanF1, 20×/0.50 objectives. Images were captured using a Spot RT3 camera with Spot software version 5.2.

### Generation of *rpoS* Mutant and Complement Strains

The strain B31 5A4 Δ*rpoS* mutant was generated by allelic exchange as previously described (Groshong et al., 2012; Supplementary Figure 1A). Briefly, upstream and downstream regions flanking *rpoS* (*bb0771*) were amplified using 5′*rpoS-*F1/3′*rpoS-*F1AscI and 5′*rpoS-*F2AscI/3′*rpoS-*F2BssHII. The upstream (F1) and downstream (F2) flanking regions were ligated together with a P*_*flgB*_-aadA*-*trpL*term streptomycin-resistance cassette from pKFSS-1 (Strep^*R*^; generously provided by Dr. Scott Samuels, University of Montana) (Frank et al., 2003) inserted into an *AscI* restriction site engineered at the F1-F2 junction. The resulting suicide vector, pJSB634A, was electroporated into B31 5A4 (Purser and Norris, 2000) as described previously (Yang et al., 2004). Streptomycin-resistant clones were screened by PCR using primer pair 5′*rpoS*diag/3′*rpoS*diag and one clone, EC3/BbP1752, containing a full complement of endogenous plasmids, was selected. For *trans* complementation, EC3/BbP1752 was transformed with a cp9-based shuttle vector (pJSB296; Supplementary Figure 1B) encoding a wild-type copy of *rpoS* expressed from its native promoter. The presence of the *trans* complement was confirmed by PCR using primer pair 5′*rpoS* diag/3′*rpoS* diag. One complemented clone, *rpoS*comp, EG13/BbP1754, was selected.

### Retention of *rpoS Trans* Complement Plasmid in Mice

Retention of empty shuttle vector (pJD44) and *rpoS trans*-complementing plasmid (pJSB259) was assessed in C3H/HeJ mice (Jackson Laboratories, Bar Harbor, ME, United States) following syringe-inoculation. Briefly, mice (15 per strain) were inoculated intradermally with 10^5^ of either BbP1974 or BbP1754. Two weeks post-inoculation (p.i.), infection was confirmed by serology and cultivation of ear tissues in BSK-II containing *Borrelia* antibiotic cocktail (BAC; 0.05 mg/ml sulfamethoxazole, 0.02 mg/ml phosphomycin, 0.05 mg/ml rifampicin, 0.01 mg/ml trimethoprim and 0.0025 mg/ml amphotericin B). Approximately 1 week prior to sacrifice, mice (3 per strain, per time point) were infested with naïve *I. scapularis* larvae as previously described (Caimano et al., 2015). Ten replete larvae per mouse (3 mice per strain, per time point) were pooled, surface sterilized and plated for single colonies on pBSK containing BAC as previously described (Caimano et al., 2011). At 4, 8, 12, 16, and 20 weeks p.i., mice were euthanized and blood and tissues were collected for serology and culture in BSK-II without kanamycin, respectively; at all time points examined, effort was made to collect similarly sized samples of each tissue from infected mice. Cultures were monitored weekly by dark-field microscopy for up to 8 weeks. As soon as cultures became dark-field positive, aliquots of ear and skin cultures were plated on pBSK without kanamycin. Agarose plugs containing individual colonies (10 per tissue or larval pool from each of 3 mice per strain, per time point) were placed in sterile microfuge tubes containing 100 μL PCR-grade water and two 1.0 mm silicon carbide beads (BioSpec Products, Inc., Bartlesville, OK, United States), heated at 80°C for 10 min, immediately transferred to a Disruptor Genie and then mixed at 3000 rpm for 2 min. Ten μL of each disrupted colony was screened for the kanamycin-resistance cassette (kan^*R*^) by PCR using primers aph-F-349 and aph-R-767. Samples testing negative for the 419-bp kan^*R*^ amplicon were re-tested using primers flaB-453-F/flaB-993-R to ensure that each contained sufficient spirochetal DNA. Statistical significance of differences was determined with GraphPad Prism (version 7) using a two-tailed *t-*test, with *p* ≤ 0.05 considered significant.

### ELISA Assay

Sera from infected mice was tested in triplicate by indirect ELISA against recombinant OspA, OspC, DbpA, BBA62, and FlaB as described previously (Gomes-Solecki et al., 2000). One hundred microliters of purified recombinant protein (0.5 μg/ml) was added to each well, and the plate was incubated overnight at 4°C. Wells were blocked for 30 min. with 200 μl of blocking solution (2% bovine serum albumin in 140 mM sodium carbonate, pH 9.0) at 37°C then washed three times with sodium carbonate, pH 9.0. One hundred microliters of serum from individual mice, diluted with specimen diluent (10% fetal bovine serum in PBS), was added to each well and the plate was incubated for 1 h at 37°C. Bound antibody was detected using goat anti-mouse IgG horseradish peroxidase-conjugated secondary antibody (Southern Research, Birmingham, AL, United States) diluted 1:1000 in specimen diluent followed by detection with BD TMB Substrate for 5–15 min at 37°C. Statistical significance between sera collected at the same time point p.i. was determined in GraphPad Prism (v. 7) using a two-tailed *t*-test with *p* ≤ 0.05 considered significant.

### Comparative RNA-Seq

Total RNAs were prepared from at least three biological replicates per strain, per condition. Samples were derived from spirochetes cultivated in rat peritoneal DMCs harvested 12–14 days post-implantation as described previously (Akins et al., 1998; Caimano, 2005). Ribosomal RNA was removed using the Ribo-Zero rRNA Removal Magnetic Kit for Bacteria (Illumina, San Diego, CA, United States). rRNA-subtracted samples were used to generate indexed paired-end libraries according to the TruSeq low-throughput protocol (Illumina). Libraries were quantified using the KAPA Library Quantification kit according to manufacturer’s instructions (KAPA Biosystems, Boston, MA, United States) and run on a 500-cycle Illumina MiSeq Reagent kit (v2), yielding ∼2 million reads per library. Raw read data for B31 were mapped with EDGE-pro (Magoc et al., 2013) using fasta, protein translation table (ptt) and ribosomal/transfer RNA table (rnt) files from the strain B31 RefSeq reference genome. Raw read data for 297 strains were analyzed using custom genome, ptt and rnt files, provided on request. Because the strain 297 strain used for genomic sequencing is missing lp25, the corresponding plasmid from the closely related strain JD-1 was used for mapping (Casjens et al., 2012). Pseudogenes and genes encoding open reading frames <60 amino acids were excluded from the ptt files for both strains. Differential expression between various conditions/time points was determined using DESeq2 (Love et al., 2014). A gene was considered RpoS-regulated if expression in the mutant differed ≥3-fold from both the wild-type and complemented strains with a False Discovery Rate (FDR)-adjusted *p*-value (*q*-value) ≤ 0.05. Raw read data and process files have been deposited in the NCBI Sequence Read Archive (SRA) database (SUB5592924 for B31 *in vitro* and DMC data and SUB5736827 for 297 DMC data).

### Bioinformatics

Routine and comparative sequence analyses were performed using MacVector (version 17.0.1, MacVector, Inc., Cary, NC, United States). Conserved domain searches were performed using CDD Search^1^. Candidate lipoproteins were identified based on Setubal et al. (2006); lipoprotein localization was based on Dowdell et al. (2017). OMPeome analysis was based on Kenedy et al. (2016). Putative RpoS homologs used for phylogeny studies were obtained by searching the SEED genome database (Disz et al., 2010). Multiple sequence alignments used for phylogenic trees were generated in Clustal Omega (Sievers et al., 2011) with default settings. Unrooted Neighbor-joining trees were visualized and annotated using Interactive Tree of Life (iTOL, v 4.3) (Letunic and Bork, 2016).

### Screening of Transposon Mutants

*Borrelia burgdorferi* strain B31 transposon (Tn) mutants were obtained from BEI Resources^2^. Individual mutants were plated on pBSK semi-solid medium to obtain single colonies. Cloned mutants were tested by PCR to confirm (i) the location of the Tn within the designated locus and (ii) the presence of the gentamicin-resistance cassette (Pless Gent F/Pless Gent R). The endogenous plasmid content of wild-type and mutant strains was confirmed by PCR as previously described (Eggers et al., 2002). Murine infection studies (3 mice per group, per time point) were performed in C3H/HeJ mice as described above using a dose of 10^4^.

### Complementation of *bba34* Transposon Mutant

The *bba34tn* mutant (BbAG104) was complemented in *cis* with a wild-type copy of *bba34* by allelic replacement in the native locus. The complementation construct was generated by amplifying fragment 1 (5′ bba34c F1/3′ bba34c F1) and fragment 2 (5′ bba34c F2/3′ bba34c F2) from B31 5A18 NP1 DNA and the *aadA* marker (5′ bba34 Strep/3′ bba34 Strep) from pKFSS-1 using CloneAmp HiFi PCR Premix (Clontech). Fragments were ligated into BamHI-digested pUC19 using InFusion EcoDry Cloning Kit (Clontech). A single clone (pEcAGAG143) was sequenced and transformed into the *bba34* Tn mutant (BbAG104). A single complemented clone (BbAG135) containing a wild-type copy of *bba34* and a full (parental) plasmid content was selected. Murine infection studies (5 mice per group, per time point, per experiment) were performed in C3H/HeJ mice as described above using a dose of 10^4^.

## Results

### Structural Modeling Confirms That BB0771 Is a RpoS Homolog

Some authorities have questioned whether BB0771 is correctly annotated as RpoS (RpoS_Bb_) (Chiang and Schellhorn, 2010; Hengge, 2011). Based on phylogeny and genomic synteny, Chiang and Schellhorn (2010) postulated that *bb0771* arose from a duplication event that is distinct from the *rpoD → rpoS* duplication that gave rise to RpoS in γ-proteobacteria (Lonetto et al., 1992). Hengge (2011) noted that BB0771 lacks (i) a conserved lysine (K173) used by *E. coli* RpoS (RpoS_Ec_) to recognize a cytidine at position -13 in RpoS-dependent promoters and (ii) a C-terminal 16 amino acid extension that interacts with the RNA polymerase core β-subunit flap domain.

**FIGURE 1:**
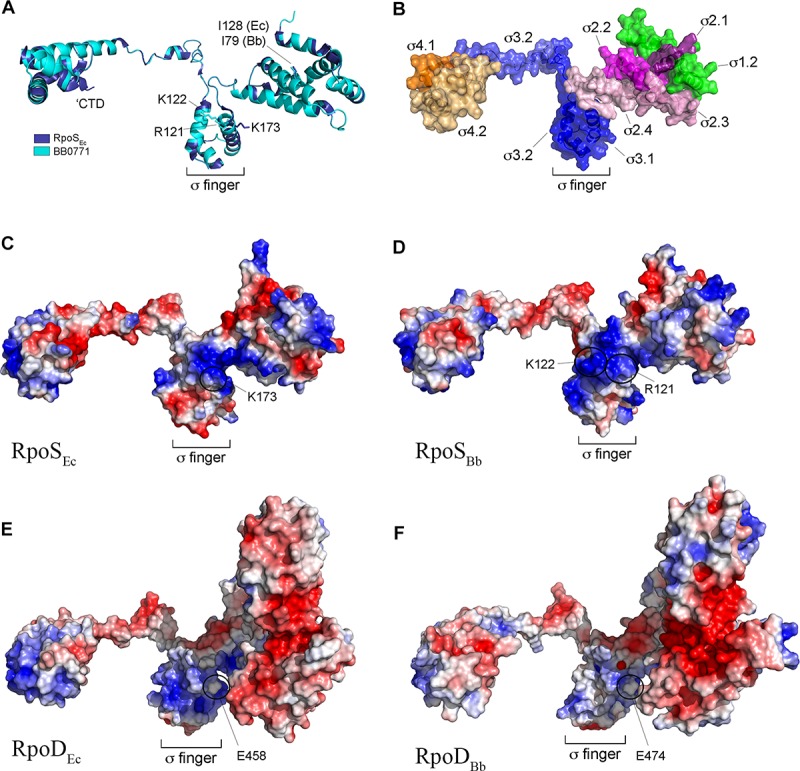
RpoS_Bb_ (BB0771) is a divergent RpoS that retains residues and structural features important for RpoS function and promoter selectivity. **(A)** Overlay of RpoS_Ec_ structure (PDB: 5IPL-F) (Liu et al., 2016) and RpoS_Bb_ homology model. Indicated are conserved isoleucine (I128 and I79 in RpoS_Ec_ and RpoS_Bb_, respectively) (Iwase et al., 2017) and positively charged residues (K173 in RpoS_Ec_ and R121 and K122 in RpoS_Bb_) (Barne et al., 1997) known or predicted to be important for promoter selectivity by RpoS. The C-terminal domain (CTD, residues 315–330 in RpoS_Ec_) conserved in RpoS homologs from γ-proteobacteria is indicated (Liu et al., 2016). **(B)** Functional subdomains in the RpoS_Bb_ homology model were defined according to the corresponding regions in RpoS_Ec_ (Lonetto et al., 1992) using the aligned structures in panel **A**. **(C–F)** Electrostatic distribution in RpoS_Ec_ **(C)**, RpoS_Bb_ homology model **(D)**, RpoD_Ec_ (PDB: 6CY9) **(E)**, and RpoD_Bb_ (BB0712) homology models **(F)**. The negatively charged glutamic acid residue (E458 in RpoD_Ec_ and E474 in RpoD_Bb_) in σ region 3.1 used to distinguish between RpoD (σ^70^) housekeeping and RpoS σ factors (Barne et al., 1997) is indicated.

We began our assessment of RpoS_Bb_ by performing an updated phylogenetic analysis of RpoS homologs available in the SEED genome database (Disz et al., 2010). As expected, almost all known or putative RpoS orthologs from γ-proteobacteria clustered tightly (Supplementary Figure 2). RpoS_Bb_, on the other hand, groups more broadly with homologs from taxonomically diverse bacteria, including β-, Δ- and ζ-proteobacteria. To gain a deeper appreciation for the origin and function of BB0771, we used I-Tasser (Roy et al., 2010; Yang and Zhang, 2015) to generate a structural homology model. Although lacking the C-terminal extension, RpoS_Bb_ modeled extremely well (C-score 1.54, TM-score 0.93 ± 0.06, RMSD 2.9 ± 2.1) against RpoS_Ec_ (Figure 1A). Importantly, using the aligned structures, we could readily identify the functional domains shared by σ^70^ family members (Lonetto et al., 1992; Figure 1B) as well as a widely conserved isoleucine residue (I128 in RpoS_Ec_ and I79 in RpoS_Bb_) critical for activity in *E. coli* (Iwase et al., 2017; Figure 1A). Promoter recognition by RpoS in *E. coli* is mediated by an interaction between a cytidine located at position -13 (C-13) of an extended -10 domain and a conserved lysine residue (K173) located within σ region 3.1 (σ_3.1_) (Becker and Hengge-Aronis, 2001; Liu et al., 2016; Figures 1A,C); the *E. coli* σ^70^ housekeeping σ factor (RpoD_Ec_), in contrast, contains a negatively charged residue (E458) at this position (Figure 1E). Like RpoS_Ec_, RpoS_Bb_ also uses an extended -10 region for promoter recognition (Eggers et al., 2006), although the discriminator nucleotides (T-15 for P*ospC* and T-13/C-15 for P*ospF*) are positioned similarly or slightly upstream from the C-13 discriminator used by RpoS_Ec_ (Becker and Hengge-Aronis, 2001; Eggers et al., 2004). Electrostatic analysis of RpoS_Bb_ revealed two positively charged surface residues (R121 and K122) positioned similarly to K173 in σ_3.1_ of RpoS_Ec_ (Figures 1C,D), either or both of which could contribute to promoter selectivity. Like RpoD_Ec_, RpoD_Bb_ contains a glutamic acid (E474) residue in the region of used for promoter selectivity by RpoS (region σ_3.1_) (Figure 1F). These data, along with our previous demonstration that RpoS_Bb_ can promote transcription from an *E. coli* RpoS-dependent promoter (P*osmY*) when expressed in an *E. coli* Δ*rpoS* mutant (Eggers et al., 2004, 2006), enable us to conclude that RpoS_Bb_ is a *bona fide*, albeit divergent, RpoS homolog.

### *ospA* Is Strongly Expressed Throughout the Tick Phase of the Enzootic Cycle

**FIGURE 2:**
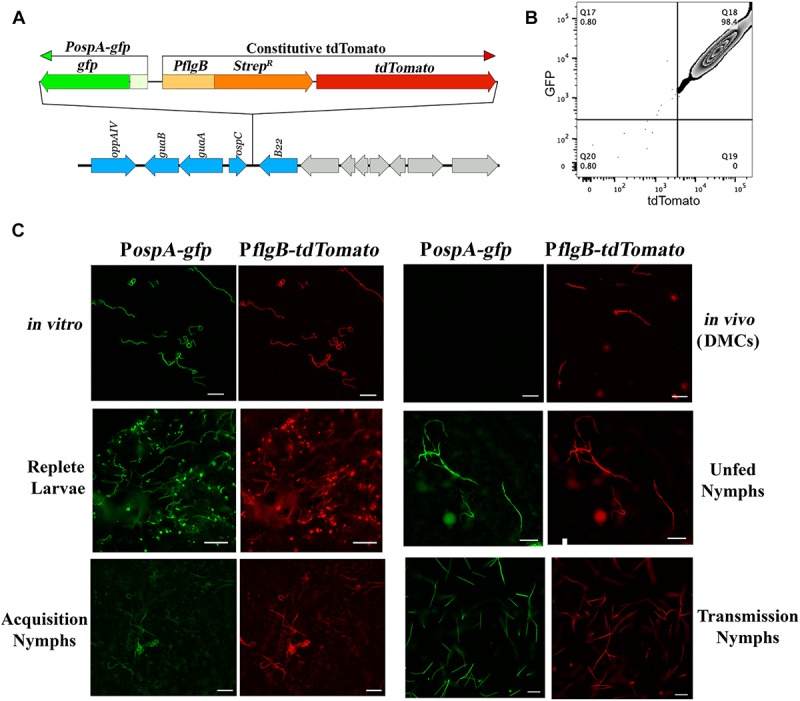
*ospA* is expressed throughout the entire tick phase of the enzootic cycle. **(A)** Cartoon depicting the tandem insertion of a P*ospA-gfp*/P*flgB tdTomato* “dual color” cassette into the cp26 *bbb21-22* intergenic region (Kawabata et al., 2004); the resulting *B. burgdorferi* strain was designated BbP1981. The upstream region used to monitor expression of *ospA* (P*ospA*) includes sequences required for RpoS-mediated repression of the *ospA* in mammals (Grove et al., 2017). **(B)** Representative quad-plots for temperature-shifted *in vitro* P*ospA-gfp* +/tdTomato+ double-positive spirochetes. Numbers indicate the percentage of positive events in each quadrant. **(C)** Representative epifluorescence images of the P*ospA-gfp*/P*flgB-tdTomato* dual color strain (BbP1981) following temperature-shift at 37°C *in vitro*; cultivation in DMCs; in *I. scapularis* larvae fed to repletion on BbP1981-infected C3H/HeJ mice; in unfed nymphs; in BbP1981-infected nymphs fed for 72–96 h on a naïve C3H/HeJ mice (Transmission Nymphs); and in naïve nymphs fed for ∼36–48 h on C3H/HeJ mice 4 weeks post-infection (Acquisition Nymphs). Scale bar, 20 μm.

As noted earlier, there are divergent viewpoints as to whether *ospA* is downregulated within nymphal midguts during transmission (Schwan et al., 1995; Pal et al., 2000, 2001, 2004; Belperron and Bockenstedt, 2001; Stewart and Rosa, 2018). To examine how the midgut environment influences expression of *ospA* during the blood meal, we developed a ‘dual color’ reporter system for monitoring transcription of *ospA* by individual spirochetes. The reporter strain used for these studies, BbP1981, expresses a well-characterized P*ospA-gfp* reporter (Eggers et al., 2002, 2006; Dunham-Ems et al., 2009, 2012; Harman et al., 2012; Grove et al., 2017) and a constitutive *tdTomato* under the control of the *flgB* promoter (P*flgB-tdTomato*) tandemly inserted in opposite orientations into cp26 (Figure 2A). Importantly, the P*ospA-gfp* reporter used for these studies mirrors the expression profile of the native lp54-encoded gene when the reporter was located on a cp9-based shuttle vector or inserted on to cp26 (Eggers et al., 2002, 2006; Dunham-Ems et al., 2009, 2012; Harman et al., 2012; Grove et al., 2017). At the outset, we confirmed the virulence of BbP1981 by needle-inoculation of C3H/HeJ mice. By 2 weeks post-inoculation (p.i.), all mice (*n* = 5) inoculated with 10^4^ BbP1981 seroconverted and yielded positive cultures for multiple tissues (ear, skin, tibiotarsal joints and bladder). Using flow cytometry and epifluorescence microscopy, we demonstrated that essentially all tdTomato+ spirochetes grown *in vitro* following temperature-shift were strongly GFP+ (Figures 2B,C), confirming at the single cell level that activation of the RpoN/RpoS pathway *in vitro* does not promote downregulation of OspA. However, as with the native *ospA* during cultivation within DMCs (Akins et al., 1998; Caimano et al., 2007, 2015; Iyer et al., 2015), the P*ospA*-*gfp* reporter was repressed while the P*flgB-tdTomato* reporter was well expressed (Figure 2C).

We next used the dual color reporter system to track expression of *ospA* in midguts when the RpoN/RpoS pathway is either OFF (acquiring and unfed nymphs) or ON (tick-to-mammal transmission) (Caimano et al., 2005, 2015; Dunham-Ems et al., 2012; Ouyang et al., 2012; Radolf et al., 2012; Iyer et al., 2015). As shown in Figure 2C, all tdTomato+ spirochetes observed in larvae fed to repletion on BbP1981-infected mice were GFP+. Expression of the P*ospA-gfp* reporter by all tdTomato+ spirochetes also was observed in the resulting unfed nymphs post-molt (Figure 2C). BbP1981-infected nymphs were allowed to feed on naïve mice for at least 72 h, a time point during tick feeding when RpoS-upregulated genes required for transmission are expressed and functional (Mulay et al., 2009; Dunham-Ems et al., 2012; Ouyang et al., 2012; Iyer et al., 2015). As with replete larvae, all tdTomato+ spirochetes observed within engorged nymphs were P*ospA-gfp+* during transmission (Figure 2C). Collectively, these findings demonstrate that *ospA* is expressed throughout the entire tick phase of the enzootic cycle, regardless of whether RpoS is in an ON or OFF state.

### Repression of *ospA* Occurs Upon Murine Infection and Continues Until Acquisition

**FIGURE 3:**
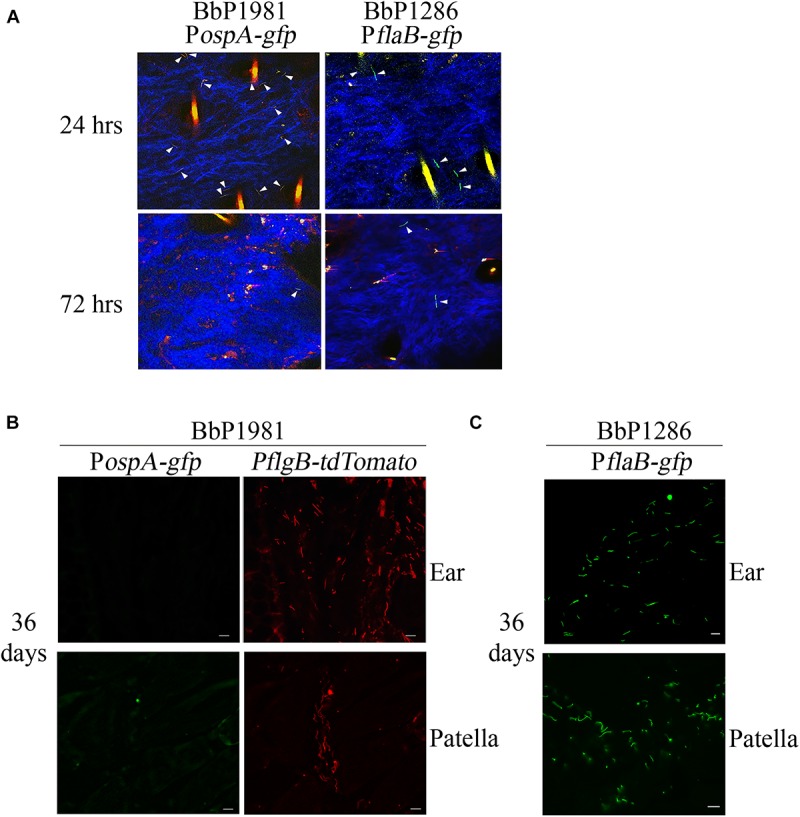
Repression regulation of *ospA* in mice is not complete until > 72 h after syringe-inoculation and continues throughout the mammalian phase. **(A)** Representative composite two-photon microscopy images of ear tissues from *Myd88*^–/–^ mice 24 and 72 h after needle-inoculation with either P*ospA-gfp* (BbP1981) or P*flaB-gfp* (BbP1286). Arrowheads indicate intact GFP+ spirochetes. Three-dimensional *z*-stack images were rendered using Volocity software from images of sequential *x, y* planes taken at different levels. Hair follicles and dermal collagen fibers fluoresce yellow-orange and blue, respectively, due to second-harmonic generation. A minimum of 20 fields per tissues were examined. Representative images for day 8 and 13 are shown in Supplementary Figure 4A. **(B,C)** Representative epifluorescence images of 7 μm cryosections of ear and patella collected from *Myd88*^–/–^ mice 36 days post-infection (p.i.) by needle with BbP1981 to detect P*ospA-gfp* and P*flgB-tdTomato* **(B)** or BbP1286 to detect P*flaB-gfp* **(C)**. Scale bar, 20 μm.

We next conducted experiments in mice to compare regulation of *ospA* in the arthropod and mammalian environments. Despite numerous attempts, investigators have been unable to visualize spirochetes in the bite site during tick feeding using intravital imaging (Bockenstedt et al., 2014), presumably due to the small numbers of organisms transmitted by feeding nymphs (Coleman et al., 1995; Ohnishi et al., 2001; Dunham-Ems et al., 2009). We were unable to use tdTomato to reliably track spirochetes in tissues because the wavelength required for optimal excitation of tdTomato (≅1050 nm) lies at the upper limit of Ti-Sapphire lasers (Drobizhev et al., 2011). Instead, following intradermal inoculation, we compared expression of GFP from the P*ospA-gfp* reporter in BbP1981 with BbP1286, encoding a well-characterized isogenic strain harboring a constitutive P*flaB-gfp* reporter (Eggers et al., 2004; Dunham-Ems et al., 2012; Caimano et al., 2015; Iyer et al., 2015). Prior to infection studies, we confirmed by flow cytometry that the P*ospA-gfp* and P*flaB-gfp* reporters express virtually identical percentages of GFP+ organisms (98.90% + 2.125 vs. 98.95% ± 0.091, respectively) and highly similar mean fluorescence intensities (26,573 + 2,033 vs. 32,007 + 11,127, respectively; *p* = 0.69) (Supplementary Figure 3). By 2-photon laser-scanning microscopy at 24 h, we observed comparable densities of GFP+ organisms in the inoculation sites of mice infected with either BbP1981 or BbP1286 (Figure 3A). BbP1286 spirochetes expressing P*flaB-gfp* were observed near the inoculation site up to 13 days p.i. (Figure 3A and Supplementary Figure 4A). In contrast, by 72 hrs p.i., we saw few P*ospA*-GFP+ BbP1981 organisms around the inoculation site (Figure 3A) and no P*ospA-gfp*+ organisms at 8 and 13 days p.i. (Supplementary Figure 4A).

To confirm at the single spirochete level that *ospA* continues to be repressed following dissemination, ear and patella tissues collected from BbP1981-infected mice 36 days p.i were embedded in OCT compound, cryosectioned and imaged by epifluorescence microscopy (which enables visualization of tdTomato as well as GFP). As shown in Figure 3B, tdTomato+ organisms were readily detected in both tissues but none were P*ospA-gfp*+. To ensure that GFP fluorescence was not substantially diminished by processing, control cryosections of ear and patella tissues from BbP1286-infected mice prepared in the same manner were processed and imaged in parallel (Figure 3C).

Lastly, we asked whether de-repression of *ospA* occurs in the skin as spirochetes migrate toward the bite site during acquisition or only after spirochetes have entered the tick midgut. No P*ospA-gfp*+ organisms were observed in or around the feeding site when naïve nymphs were fed to repletion (∼96 hrs) on Bb1981-infected mice (Supplementary Figure 4B). In contrast, as noted above (Figure 2C), engorged, acquiring nymphs contained numerous P*ospA-gfp*+/tdTomato+ double-positive spirochetes. To establish the time frame for de-repression of *ospA* during acquisition, nymphs were forcibly removed from BbP1981-infected mice beginning 24 hrs post-placement and their midguts examined. As early as 36 hrs post-placement, all tdTomato+ BbP1981 spirochetes detected in partially fed nymphal midguts were P*ospA-gfp*+ (Figure 2C).

### BosR-Dependent Repression of *ospA* Within the Mammalian Host Requires the RpoN/RpoS Pathway

**FIGURE 4:**
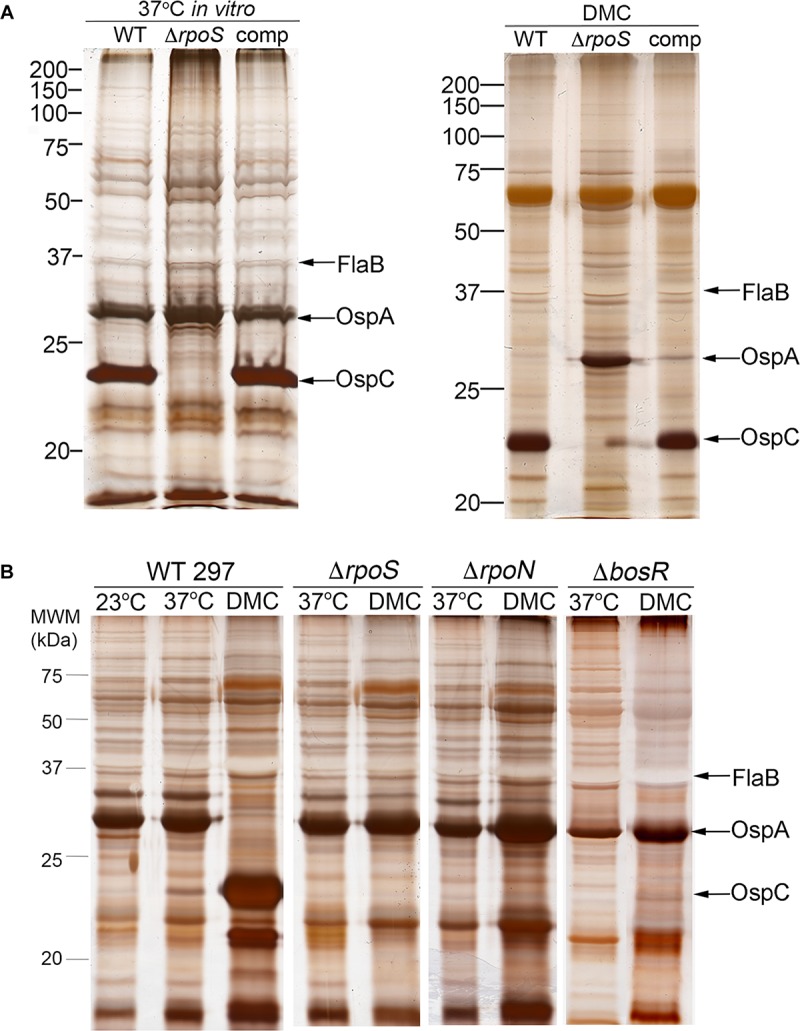
Reciprocal regulation of OspC and OspA by RpoS occurs only within mammals and requires an intact RpoN/RpoS pathway. **(A)** Whole-cell lysates from wild-type strain B31 5A4 (WT; BbP1781), isogenic Δ*rpoS* mutant (BbP1752) and complemented mutant (comp; BbP1754) strains following temperature-shift *in vitro* and cultivation in DMCs separated by SDS-PAGE and stained with silver. **(B)** Whole cell lysates of wild-type strain 297 and isogenic Δ*rpoS*, Δ*rpoN*, and Δ*bosR* mutants following temperature-shift from 23 to 37°C *in vitro* and cultivation in DMCs. Molecular weight markers (kDa) are shown at the left of each gel.

Shi et al. (2014) reported that BosR, a Fur/PerR-like *trans*-acting factor that partners with RpoN to transcribe *rpoS* (Boylan et al., 2003; Hyde et al., 2009; Ouyang et al., 2009), can repress transcription of *ospA* by binding to *cis* elements within the gene’s promoter region. Their data, however, were generated by *in vitro* cultivation of a B31 strain that overexpresses BosR. Using strain 297 Δ*rpo*S mutant and complement, we previously demonstrated that repression of *ospA* within mammals (i.e., cultivation in DMCs) does not occur in the absence of RpoS (Caimano et al., 2005; Dunham-Ems et al., 2012; Grove et al., 2017). Herein, we confirmed these findings for strain B31 using isogenic wild-type 5A4, Δ*rpo*S and *trans-*complemented isolates (BbP1781, BbP1752, and BbP1754, respectively) (Figure 4A). Previously, Wang et al. (2013) demonstrated that a strain B31 Δ*bosR* mutant failed to downregulate OspA within DMCs. To confirm and extend these findings, we took advantage of existing Δ*bosR*, Δ*rpoN*, and Δ*rpoS* isogenic mutants (Caimano et al., 2004, 2005; Ouyang et al., 2008, 2009) to determine whether BosR can repress *ospA* independently of RpoN under biologically relevant conditions. As expected, none of the strain 297 isogenic mutants expressed OspC *in vitro* or within DMCs (Figure 4B), underscoring the well-established requirement of BosR and RpoN for expression of *rpoS* and RpoS-upregulated genes (Hubner et al., 2001; Fisher et al., 2005; Hyde et al., 2009; Ouyang et al., 2009). In contrast to the wild-type 297 parent, none of the mutants downregulated OspA in DMCs (Figure 4B). Thus, repression of OspA in mammals requires an intact RpoN/RpoS pathway.

### RpoS Is Required for Persistence of Spirochetes in Mice

While *rpoS* and some RpoS-upregulated genes (e.g., *dbpA*, *bba65*, and *bba66*) are expressed throughout the mammalian host phase (Liang et al., 2002a; Gilmore et al., 2008; Ouyang et al., 2012), no study to date has looked at the contribution of this alternative σ factor to persistence. Toward this end, we performed a modified version of the plasmid retention approach used by Tilly et al. (2006) to establish that *ospC* is required only during early infection. Their study demonstrated that complementation of an Δ*ospC* mutant in *trans* was essential for murine infection *via* needle-inoculation; by 28 days p.i., almost all spirochetes recovered from infected tissues had lost the *ospC*-complementing plasmid. Positive selection for Δ*ospC* spirochetes lacking the complementing plasmid was driven by the adaptive immune response (Tilly et al., 2006). Using an analogous approach, we measured retention of the *rpoS*-complementing plasmid in the B31 *rpo*Scomp strain (BbP1754). At the outset, we confirmed that *trans*-complementation restores virulence of the mutant by both needle- and tick-inoculation (Supplementary Table 3). We next compared the persistence of BbP1754 with the wild-type parent transformed with empty shuttle vector (BbP1974) over a 20-wk time course. Recovery of viable spirochetes from tissues (ear, distal skin, tibiotarsal joints and heart) of mice infected with either strain was scored cumulatively based on (i) the number of tissues from which viable spirochetes were recovered and (ii) the time required for individual tissue cultures to become darkfield-positive (Table 1); these same data are displayed graphically in Supplementary Figure 5. All but two (1 of 3 hearts at 12 weeks and 1 of 3 tibiotarsal joints at 20 weeks) of the 60 individual tissues collected from BbP1974-infected mice were culture-positive; the average cumulative scores for all tissues at each time point remained high (>2.5) for up to 16 weeks, dropping only slightly at 20 weeks. In contrast, tissues collected from BbP1754-infected mice scored much lower overall. At 4 weeks, 3 of 3 hearts collected from BbP1754-infected mice were culture-negative, while remaining tissues were positive. At 8, 16, and 20 weeks, the cumulative culture scores for BbP1754 were significantly (*p* ≤ 0.05) lower compared to the same time point for BbP1974-infected mice; culture scores for BbP1754 also trended lower at 12 weeks but were not significantly different (*p* = 0.069) from BbP1974 at the same time point. Notably, at 20 weeks, only ear (3/3) and skin (1/3) tissues from BbP1754-infected mice were culture-positive.

**TABLE 1:** Expression of *rpoS* and RpoS-regulated genes is required by *B. burgdorferi* for persistence in mice.

**Strain^1^**	**Mouse^2^**	**4 weeks^3^**				**8 weeks**				**12 weeks**				**16 weeks**				**20 weeks**			
		
		**ear**	**skin**	**joint**	**heart**	**ear**	**skin**	**joint**	**heart**	**ear**	**skin**	**joint**	**heart**	**ear**	**skin**	**joint**	**heart**	**ear**	**skin**	**joint**	**heart**
WT+empty vector^1^ (BbP1974)	1	+++	++	++	++	++	++	+++	+++	+++	+++	+++	–	+++	+++	+++	++	++	++	–	++
	2	+++	+++	+++	+++	+++	+++	+++	++	+++	+++	+++	+++	+++	++	+++	++	++	++	++	++
	3	+++	++	+++	++	+++	+++	+++	+++	+++	+++	+++	+++	++	+++	+++	++	++	++	++	++
*rpoS*comp (BbP1754)	1	++	++	++	–	++	–	–	–	+++	+++	+++	–	++	++	–	++	++	–	–	–
	2	+++	++	+++	–	++	++	++	+	+++	–	+++	+	+++	++	++	–	++	++	–	–
	3	+++	+++	++	–	++	++	++	–	–	+++	++	–	++	++	++	++	++	–	–	–

*^1^Wild-type B31 5A4 transformed with an empty cp9 shuttle vector; *rpoS*comp, B31 5A4 Δ*rpoS* mutant complemented in *trans* with a wild-type copy of *rpoS* on a cp9-based shuttle vector. ^2^3 mice per strain, per time point. ^3^Cultures were checked at least once weekly by darkfield microscopy for the appearance of spirochetes. Plus (+) signs are used to indicate the time required for cultured tissues to become positive for spirochetes by darkfield microscopy: (+++), 1–2 weeks; (++) 3–4 weeks; or (+) 4–5 weeks post-collection. Negative symbol (−) indicates tissues that were culture-negative > 5 weeks post-collection.*

**FIGURE 5:**
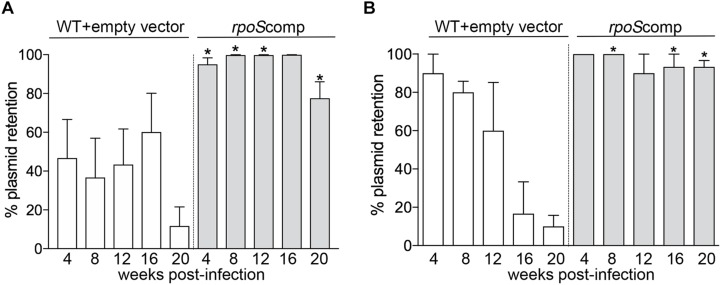
RpoS is required for persistence in mice. Retention of empty vector or *rpoS*-complementing plasmid by BbP1974 (WT+empty vector) and BbP1754 (*rpoS*comp) strains in tissues collected from infected mice 4, 8, 12, 16, and 20 weeks post-infection. Retention was assessed by PCR of individual colonies obtained by semi-solid phase plating of **(A)** murine ear and skin minimally cultured in BSK-II without antibiotic and **(B)** larvae fed to repletion on infected C3H/HeJ mice at 4 week intervals beginning 4 weeks post-infection. Columns represent the average of at least 10 colonies per cultured tissue per time point. Numbers on *x*-axis indicate the number of weeks post-infection. *p*-Values for pairwise comparisons (WT+empty vector and *rpoS*comp at the same time point) were determined using a two-tailed *t*-test; ^∗^*p* ≤ 0.05.

To compare retention of the *rpoS*-complementing plasmid and empty vector, spirochetes recovered from BbP1754- and Bb1974-infected mice were plated on semi-solid medium without kanamycin, and individual colonies were tested for the presence of the kanamycin-resistance marker by PCR. As shown in Figure 5A, for weeks 4–16 p.i., ∼40–60% of spirochetes recovered from ears and skin, respectively, of BbP1974-infected mice contained the empty vector; retention dropped to ∼10% at 20 weeks. In contrast, up to 16 weeks p.i., 80–100% of spirochetes recovered from the ears and skin, respectively, of BbP1754-infected mice contained the *rpoS*-complementing plasmid, dropping only slightly to 78% at 20 weeks (Figure 5A). Percent retention values for the *rpoS*-complementing plasmid were significantly (*p* ≤ 0.05) higher compared to empty vector at all time points except 16 weeks (*p* = 0.073). Lastly, we compared plasmid retention at each time point in larvae fed to repletion on BbP1974 or BbP1754-infected mice. As shown in Figure 5B, we observed progressive loss of the empty vector by BbP1974 in larvae over time, while the *rpoS*-complementing plasmid was retained by ≥80% of BbP1754 spirochetes over the 20-week time course; significantly (*p* ≤ 0.05) higher retention of the *rpoS-*complementing plasmid compared to empty vector was observed at 8, 16, and 20 weeks.

### Infection With Spirochetes Lacking RpoS Results in the Production of Antibodies Against OspA and Lp6.6

**FIGURE 6:**
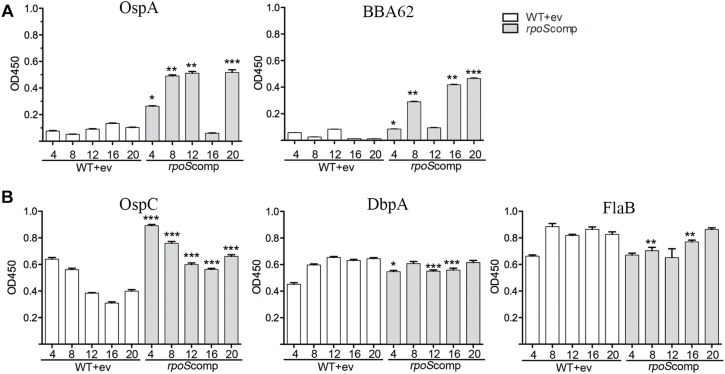
Loss of the *rpoS*-complementing plasmid by Δ*rpoS B. burgdorferi* is associated with production of antibodies against OspA and BBA62 in mice. ELISA assay performed using sera collected from individual C3H/HeJ mice and purified recombinant proteins for tick-phase gene products (OspA and BBA62) repressed by RpoS in mammals **(A)** and genes products upregulated (OspC and DbpA) or unaffected (FlaB) by RpoS **(B)**. Sera was collected at the designated time points (in weeks) following syringe-inoculation with either WT+empty vector (BbP1974) or *rpoS*comp (BbP1754). Columns represent the average and standard error of the mean from three mice per strain, per time point (4- to 20-weeks). *p*-Values for pairwise comparisons (WT vs. *rpoS*comp) were determined using a two-tailed *t*-test. ^∗^*p* ≤ 0.05; ^∗∗^*p* ≤ 0.001; ^∗∗∗^*p* ≤ 0.0001.

Our model predicts that RpoS-mediated repression of tick-phase genes, such as *ospA*, is essential throughout the mammalian phase. If so, then loss of the *rpoS*-complementing plasmid by Δ*rpo*S should lead to transcription of RpoS-repressed tick-phase genes with consequent production of antibodies against the corresponding proteins. As shown in Figure 6A, by enzyme linked immunosorbent assay, we detected significantly (*p* ≤ 0.05) higher levels of antibodies against OspA (4, 8, 12, and 20 weeks) and BBA62/Lp6.6 (4, 8, 16, and 20 weeks) in sera from BbP1754-infected mice compared to BbP1974-infected mice. Previously, we demonstrated that increased levels of RpoS expressed under the control of an IPTG-inducible promoter had no effect on the levels of OspA or BBA62 *in vitro* (Caimano et al., 2007). We argue, therefore, that the increased levels of OspA and BBA62 within DMCs are due to de-repression of the corresponding genes following loss of the *rpoS-*complementing plasmid rather than sigma factor competition. Overall, BbP1754-infected mice produced either higher or comparable antibodies against the RpoS-upregulated gene products OspC and DbpA, as well as the RpoD-dependent flagellin (FlaB) (Figure 6B). The slightly higher levels of OspC antibody in the *rpoS*comp are likely due to increased expression of *rpoS* when expressed in *trans* on a multicopy plasmid. Collectively, these data confirm that complementation of the Δ*rpoS* mutant in *trans* restores expression of RpoS-upregulated genes to wild-type levels (Caimano et al., 2007).

### Delineation of the RpoS DMC Regulon by RNA-Seq to Identify Genes Potentially Required for Persistence

Our plasmid retention experiments imply that the RpoS regulon contains one or more genes required to sustain infection. A necessary starting point for the identification of RpoS-dependent ‘persistence genes’ is an accurate and comprehensive catalog of RpoS-regulated genes expressed within the mammal. Toward this end, we performed RNA-seq on wild-type, Δ*rpoS* and *rpoS-*complemented strains following cultivation in DMCs (Akins et al., 1998; Caimano, 2005). Stringent criteria were used to define RpoS-dependence: only genes that showed ≥3-fold regulation (*q* ≤ 0.05) in both Δ*rpoS* vs. WT and Δ*rpoS* vs. *rpoS*comp comparisons were included in the final DMC regulon (Tables 2, 3). For comparison, we also determined the RpoS *in vitro* regulon using the WT, Δ*rpoS* and *rpoS*comp strains following temperature-shift.

**TABLE 2:** Genes upregulated by RpoS in *Borrelia burgdorferi in vitro* and within DMCs.

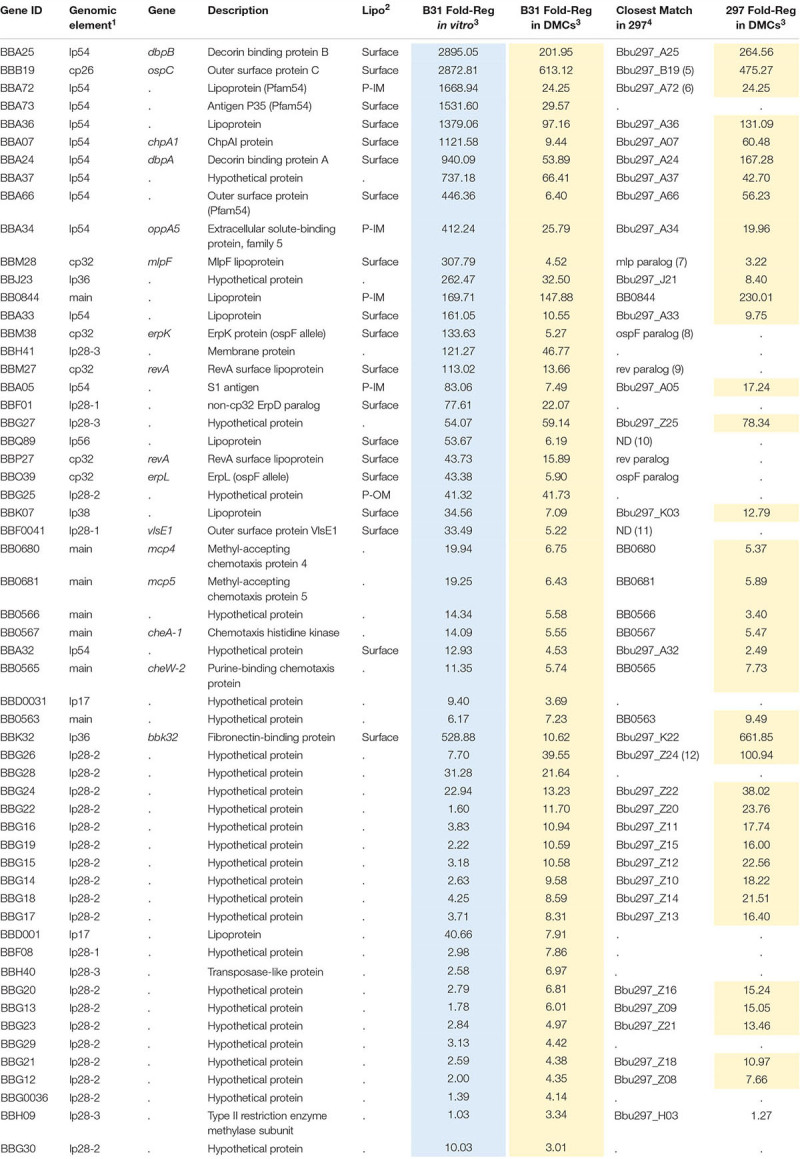

*^1^Corresponding genome location for respective genes in strains B31 and 297. ^2^Lipoprotein localization for strain B31 orthologs based on Zuckert et al. (2004) and/or previously published reports. P_IM, periplasmic leaflet of inner membrane; P-OM, periplasmic leaflet of the outer membrane. ^3^Values are for the WT vs. Δ*rpoS* mutant comparison. Highlighting is used to indicate genes that were upregulated at least 3-fold *in vitro* (blue) and/or DMCs (yellow) with adjusted *P* (*q*) value < 0.05 in wildtype vs. Δ*rpoS* mutant comparison and *rpoS*comp vs. Δ*rpoS* mutant comparisons. ^4^Closest match in strain 297 based on pairwise BLAST-P. Proteins sharing > 90% identity and located on similar genetic elements were considered orthologous. Dots (.) indicates genes for which no clear ortholog could be identified in strain 297. ^5^BBB19/OspC lipoproteins in strains B31 and 297 share 78% identity. ^6^*bba72* also referred to as *bba0078.*^7^RpoS-upregulated Mlp paralogs in strain 297 are *Bbu297_R30/mlp7b* (3.19-fold), *Bbu297_S29/mlp8* (3.45-fold), and *Bbu297_X28/mlp10* (3.22-fold). ^8^RpoS-upregulated OspF paralogs in strain 297 are *Bbu297_S41/ospF* (10.34-fold) and *Bbu297_W43/bbk2.11* (5.87-fold). ^9^Rev paralogs in strains 297 are *Bbu297_R28* (37.63-fold) and *Bbu297_X27* (19.06-fold). ^10^ND, Not detected; strain 297 does not contain a lp56 (Q) equivalent. ^11^ND, Not detected; sequence for the *vlsE1* expression site is missing from the strain 297 genome sequence. ^12^BBG26 and Bbu297_Z24 share 80% identify.*

**TABLE 3:** Genes repressed by RpoS in *Borrelia burgdorferi in vitro* and within DMCs.

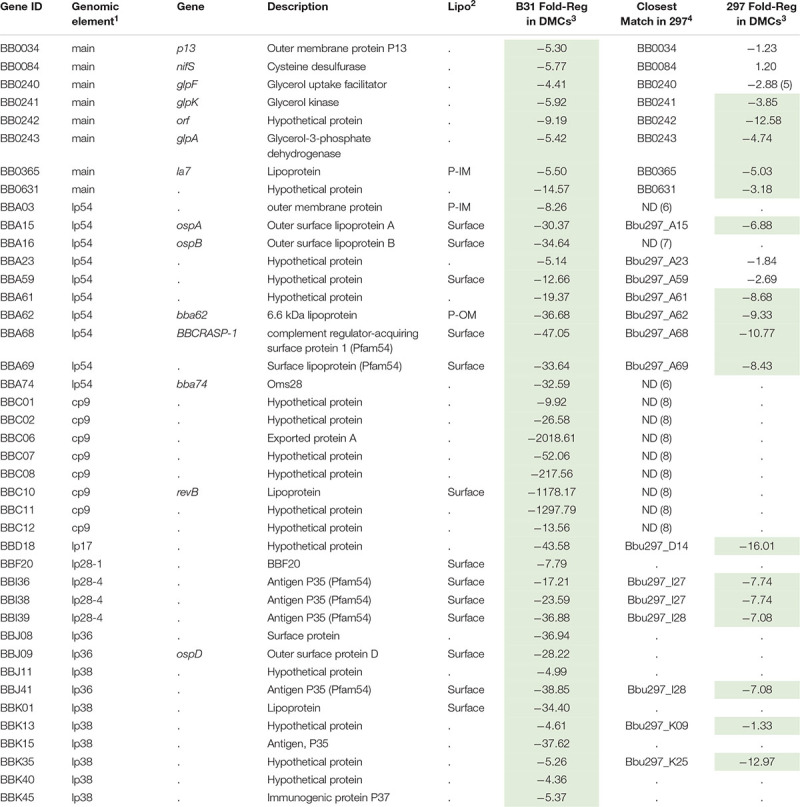

*^1^Corresponding genome location for respective genes in strains B31 and 297. ^2^Lipoprotein localization for strain B31 orthologs based on Zuckert et al. (2004) and/or previously published reports. P_IM, periplasmic leaflet of inner membrane; P-OM, periplasmic leaflet of the outer membrane. ^3^Values are for the WT vs. Δ*rpoS* comparisons. Highlighting is used to indicate genes that were repressed by RpoS at least 3-fold *in* DMCs with adjusted *p* (*q*) value < 0.05 in WT vs. Δ*rpoS* mutant comparison and *rpoS*comp vs. Δ*rpoS* mutant comparisons. ^4^Closest match in strain 297 based on pairwise BLAST-P. Proteins sharing > 90% identity and located on similar genetic elements were considered orthologous. Dots (.) indicates genes for which no clear ortholog could be identified in strain 297. ^5^RpoS-repression of *bb0240* in strain 297 has been confirmed previously by qRT-PCR. ^6^Sequences for *bba03* and *bba74*, located near the telomeric regions of lp54, are missing from the strain 297 genome sequence file used for mapping. *bba74* has been shown previously to be repressed by RpoS in strain 297 as well as B31 (Caimano et al., 2007; Mulay et al., 2009; Dunham-Ems et al., 2012). ^7^*ospB* in strain 297 contains a verified frameshift and was excluded as a pseudogene during mapping. ^8^cp9 is missing in the low-passage, virulent strain 297 isolated used for these analyses. This small circular plasmid is unstable and readily lost *in vitro*. Thus, the large folds of regulation observed for cp9-encoded genes in strain B31 could be due to plasmid loss rather than RpoS-mediated repression.*

#### Upregulated Genes

The RpoS DMC regulon contains 57 upregulated genes, the majority of which encode *Borrelia*-specific hypothetical proteins (HPs) of unknown function (Table 2). To gain insight into potential roles for these RpoS-upregulated genes in mammals, we searched the NBCI conserved domain database (CDD) (Marchler-Bauer et al., 2011) using their corresponding HP sequences; with the exception of *bb0566* and *bba07/chpA1* (see below), no notable conserved domains were identified. Twenty-two genes were upregulated by RpoS within DMCs but not *in vitro* following temperature-shift. Eighteen of these ‘DMC-only’ upregulated genes are located on lp28-2 and encode cytoplasmic HPs (Purser and Norris, 2000); all but two are transcribed in the same (minus strand) orientation and may form a large operon (Supplementary Figure 6). Thirty-five genes were upregulated by RpoS *in vitro* as well as in DMCs, including several with known or presumptive virulence-related functions – *ospC/bbb19* (Schwan et al., 1995; Tilly et al., 1997; Grimm et al., 2004; Xu et al., 2007; Skare et al., 2016; Caine et al., 2017), *dbpBA/bba25-24 (*Guo et al., 1995; Brown et al., 2001; Fischer et al., 2003; Shi et al., 2008; Weening et al., 2008), *oppA5/bba34* (Bono et al., 1998; Lin et al., 2001; Wang et al., 2004; Medrano et al., 2007; Raju et al., 2011; Groshong et al., 2017), *bbk32* (Li et al., 2006; Seshu et al., 2006; He et al., 2007; Lin et al., 2015b; Garcia et al., 2016) and the chemotaxis-related genes *bb0680-681*/*mcp4-5*, *bb0567*/*cheA1*, and *bb0565*/*cheW2* (Charon et al., 2012; Zhang et al., 2012). *bbf0041*, encoding VlsE1, a variable surface lipoprotein whose role in immune evasion has been studied extensively (Zhang et al., 1997; Bankhead and Chaconas, 2007; Norris, 2014; Verhey et al., 2018, 2019) also was upregulated by RpoS *in vitro* and within DMCs.

In *E. coli*, activation of RpoS induces a complex, coordinated adaptive response involving a large cohort of genes that render the bacterium resistant to diverse physiological and environmental stresses (Hengge, 2011). The *B. burgdorferi* RpoS DMC regulon, in contrast, contains only one upregulated gene (*bba34/oppA5*) with an obvious role in spirochete physiology; OppA5 is one of five oligopeptide substrate-binding proteins used by the ABC-type Opp transporter system (Bono et al., 1998; Groshong et al., 2017). *bb0566* is another RpoS-upregulated gene potentially related to physiological adaptation; BB0566 contains a s ulfate t ransporter and a nti-σ factor antagonist (STAS) domain shared by anti-anti-σ factors, nucleoside triphosphate binding proteins, and SulP transporters in diverse bacteria (Sharma et al., 2011). *bba07/chpA1* encodes a ∼16 kDa surface-exposed lipoprotein (Xu et al., 2010) containing a MazE-like antitoxin domain (*E*-value = 7.1e−03). In other bacteria, toxin-antitoxin modules regulate a wide range of cellular processes, including plasmid maintenance, biofilms, persistence, general stress responses, and defense against bacteriophages (Otsuka, 2016). Given the surface location of BBA07, it is unlikely to function as an antitoxin *per se* but probably promotes interactions at the spirochete-host interface.

Eight RpoS-upregulated genes belong to paralogous gene families located on cp32 (*ospE/ospF/elp*, *mlp* and *revA*) (Supplementary Figure 7A) and lp54 (Pfam54) (Supplementary Figure 8) plasmids. Although annotated as ‘Erps,’ *ospE*/*ospF*/*elp*-encoded lipoproteins fall into three evolutionarily distinct families (Supplementary Figure 7B; Akins et al., 1999; Brisson et al., 2013). Of the B31 strain’s 12 *ospE*/*ospF*/*elp* genes, only *bbm38/erpK* and *bbo39/erpL*, both *ospF*s, were upregulated by RpoS (Supplementary Figure 7B). Lin et al. (2015a) reported that ErpK and ErpL function as adhesins *via* their ability to bind heparin and/or heparin sulfate. The Mlp lipoproteins fall into two classes (Yang et al., 1999, 2003b; Porcella et al., 2000; Supplementary Figure 7C). Seven of the eight B31 *mlp*s belong to Class I. Strikingly, only one (*bbm28/mlpF*) was upregulated by RpoS. Strain B31 encodes two identical RevA surface lipoproteins, BBM27 and BBP27 (Floden et al., 2013; Byram et al., 2015), both of which were upregulated by RpoS (Supplementary Figure 7D). Of the eleven Pfam54 paralogs in strain B31 (Hughes et al., 2008; Wywial et al., 2009), three (*bba66*, *bba72* and *bba73*), all located on lp54, were upregulated by RpoS in DMCs (Table 2 and Supplementary Figure 8). BBA66, a surface lipoprotein, has been shown to be involved in tick transmission (Patton et al., 2013). *bba64*, a well characterized Pfam54 lipoprotein required for tick transmission (Patton et al., 2011), was significantly upregulated by RpoS *in vitro* (11.79-fold) but not within DMCs. Consistent with this result, Gilmore et al. (2007) found that expression of *bba64* was substantially lower levels by strain B31 in murine tissues compared to *in vitro*.

#### RpoS-Repressed Genes

Forty-one genes were downregulated by RpoS in DMCs (Table 3). As with the upregulated genes, the majority of RpoS-repressed genes encode HPs of unknown function. However, in addition to *ospA/B*, several have been examined in the context of the tick-mouse cycle. As noted earlier, gene products encoded by the *glp* operon (*bb0240-243*) are required for maximal fitness in ticks during the blood meal and molt (Caimano et al., 2011; He et al., 2011; Pappas et al., 2011). *bba62/lp6.6*, encoding a ∼7 kDa periplasmic lipoprotein, is part of a multiprotein OM-associated complex that appears to be required for persistence in ticks (Lahdenne et al., 1997; Promnares et al., 2009; Yang et al., 2013). *bbd18*, encoding a ∼26 kDa cytoplasmic protein with no obvious conserved domains, is particularly noteworthy given its proposed role in a *Borrelia*-specific pathway for targeted degradation of RpoS by the ClpXP protease during larval acquisition (Dulebohn et al., 2014; Hayes et al., 2014). *bb0365*, also referred to as LA7, encodes a 22-kDa subsurface lipoprotein required for survival in ticks during the blood meal and post-repletion (Pal et al., 2008; Yang et al., 2013). Five Pfam54 genes (*bba68*, *bba69*, *i36*, *i38*, and *i39*) (Hughes et al., 2008; Wywial et al., 2009) are repressed by RpoS in DMCs (Supplementary Figure 8). *bba68*, encoding complement-regulator acquiring surface protein 1 (BbCRASP1), is thought to contribute to complement resistance during the blood meal and very early dissemination in mammals (Barbosa and Isaac, 2018).

### *bba34/oppA5* Is a RpoS-Upregulated Persistence Gene

To identify RpoS-upregulated genes that enhance persistence in mammals, we took advantage of the strain B31 signature-tagged transposon (Tn) mutant library generated by Tao, Norris and colleagues (Lin et al., 2012). Four mutants, Δ*bba07/chpA1*, Δ*bba34/oppA5* and two Pfam54 paralog mutants, Δ*bba72* and Δ*bba73* (Supplementary Figure 9), were selected for evaluation during early infection and persistence. At the outset, individual Tn mutant clones were isolated from semi-solid medium and checked to confirm that each contained the same endogenous plasmid profile as the B31 5A18 NP1 parent. Infectivity of all four Tn mutants was compared to that of the parent in C3H/HeJ mice at 2 and 8 weeks (Table 4). None of the mutants showed decreased infectivity at the early time point. However, at 8 weeks, two (Δ*bba07* and Δ*bba34*) showed decreased culture-positivity compared to the parent.

**TABLE 4:** Tn mutant screen to identify RpoS-upregulated genes required for persistence in mice.

**Strain**	**2 weeks**		**8–9 weeks post-inoculation**					
	**Ear**	**Serology**	**Ear**	**Skin**	**Joint**	**Heart**	**Bladder**	**Total**
*wildtype*	5/5	10/10	10/10	10/10	10/10	10/10	9/10	49/50
*bba07tn*	3/3	3/3	3/3	2/3	2/3	1/3	2/3	10/15
*bba34tn*	3/3	5/5	4/5	4/5	1/5	0/5	1/5	10/25
*bba72tn*	3/3	3/3	3/3	3/3	3/3	3/3	3/3	15/15
*bba73tn*	3/3	3/3	3/3	3/3	3/3	3/3	3/3	15/15

**FIGURE 7:**
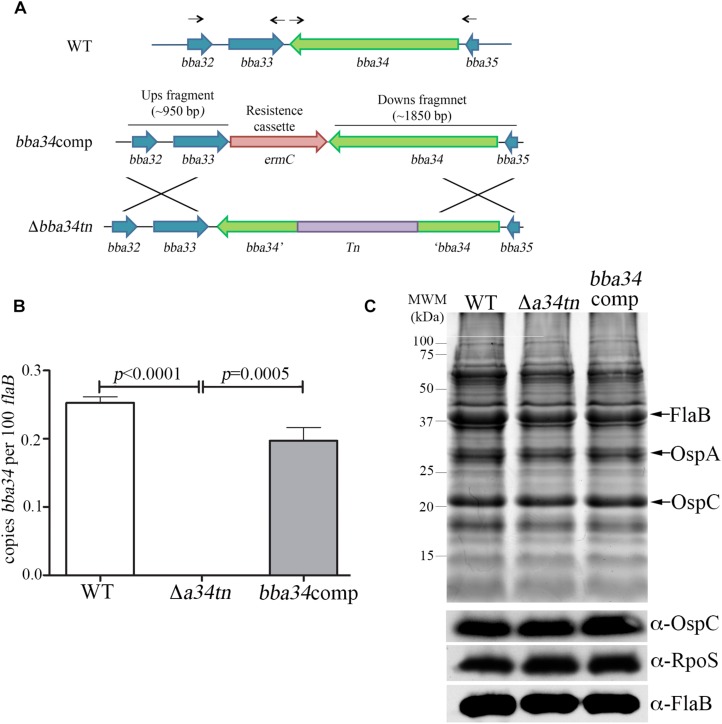
Characterization of *bba34* transposon mutant and complemented strains *in vitro.* **(A)** Cartoon schematic of wild-type (WT), *bba34* transposon mutant (Δ*a34tn*) and allelic-exchange strategy used to generate *bba34* complement (*bba34comp*). Arrows shown above the WT loci indicate location of primers used to amplify upstream and downstream fragments in *bba34*comp allelic replacement construct. qRT-PCR **(B)** and immunoblot **(C)** analyses of WT, Δ*a34tn* and *a34comp* strains following temperature-shift *in vitro*. Transcript levels for *bba34* were normalized using a TaqMan-assay for *flaB*. Error bars indicate standard errors of the mean (3 biological replicates per strain, assayed in quadruplicate). *p-*Values for pairwise comparisons were determined using a two-tailed *t*-test. Whole cell lysates were separated on a 12.5% SDS polyacrylamide gel and then either stained with Coomassie blue or transferred to nitrocellulose for immunoblotting with polyclonal antisera against strain B31 OspC (generated as part of these studies), RpoS (Hyde et al., 2007) and FlaB (Caimano et al., 2005). Molecular markers (kDa) are shown on the left.

*B. burgdorferi* relies on oligopeptides imported *via* its elaborate Opp ABC-type transporter system for growth *in vitro* and virulence in mice (Groshong et al., 2017). Of the transporter’s five differentially expressed OppA substrate-binding proteins, only *oppA5* is under RpoS control (Caimano et al., 2007; Medrano et al., 2007; Dunham-Ems et al., 2012; Table 2). Reduced culture-positivity of the Δ*bba34* Tn mutant at the 8 wk time point implies that peptide binding by OppA5 contributes to persistence in mice. To explore this further, we compared the virulence and persistence of wild-type, Δ*bba34* Tn mutant and *cis*-complemented strains (Figure 7A) in mice following syringe-inoculation (Table 5). Prior to infection studies, we confirmed by qRT-PCR that complementation restored expression of *bba34* to wild-type levels (Figure 7B). As early as 2 weeks, we observed an obvious decrease in culture-positivity for the mutant in tibiotarsal joints (3/10 mice); this defect may be due, in part, to impaired dissemination or decreased survival within joints immediately following infection. By 8 weeks, we saw reduced culture-positivity for all tissues, with tibiotarsal joints from all Δ*bba34*-infected mice (*n* = 8) being negative. Virulence and persistence of the mutant at 2- and 8-weeks were restored by *cis*-complementation (Table 5). Seshu and co-workers reported that spirochetes lacking *oppA5* dysregulate RpoS (Raju et al., 2011). Thus, it was possible that the persistence defect of the Δ*bba34* mutant was due to an indirect effect on RpoS levels. In our hands, however, inactivation of *bba34* had no effect on the expression of RpoS or RpoS-dependent genes (Figure 7C).

**TABLE 5:** BBA34/OppA5 is required for persistence in mice.

	**2 weeks^1,2^**			**8 weeks^1^**		
	**WT**	**Δ*bba34*tn**	***bba34*comp**	**WT**	**Δ*bba34*tn**	***bba34*comp**
Ear	14/14	9/10	4/4	14/14	6/8	4/4
Inoculation site	14/14	9/10	3/4	14/14	4/8	3/4
Tibiotarsal joint	14/14	3/10	4/4	14/14	0/8	4/4
Heart	14/14	8/10	4/4	14/14	4/8	4/4
Total # positive tissues	56/56	29/40	15/16	56/56	14/32	15/16
Total # infected mice	14/14	10/10	4/4	14/14	7/8	4/4

*^1^Results for represent combined data from at least two independent experiments using C3H/HeJ mice. ^2^All mice seroconverted regardless of strain or timepoint.*

### Overlap and Divergence of the RpoS DMC Regulons in Strains B31 and 297

The availability of a complete 297 genome (Casjens et al., 2012) enabled us to perform the first inter-strain comparison of RpoS DMC regulons using RNA-seq. Strains B31 and 297 differ with respect to their origins (nymph vs. human isolate), ribosomal RNA intergenic spacer lineages (1 vs. 2), and OspC (A vs. K), PFGE (B vs. A) and MLST types (1 vs. 3) (Casjens et al., 2012, 2017). Genome-wide BLAST-P searches (Altschul et al., 1990; Gish and States, 1993) were performed to identify highly conserved (≥90% amino acid identity) syntenic gene products within the two strains.

#### RpoS-Upregulated Genes With Highly Conserved Orthologs in Both Strains

Forty-three of the 57 RpoS-upregulated genes in B31 have identifiable orthologs in 297. Of these, 41 were similarly upregulated by strain 297 in DMCs (Table 2), including 14 lp28-2-encoded genes upregulated by RpoS only within DMCs (Supplementary Figure 6). Collectively, these genes likely constitute the ‘core’ regulon of genes upregulated by *B. burgdorferi* during tick transmission and/or mammalian infection. Only two orthologous genes (*bba32* and *bbh09*) were upregulated by RpoS in B31 but not 297 (Table 2). Fourteen genes upregulated by RpoS in B31 had no obvious ortholog (or paralog) in 297. Two of these, *bba73* and *vlsE1*, are located near telomeres and missing from the strain 297 NCBI GenBank entries for lp54 and lp28-1 used for read mapping (Casjens et al., 2017). The remaining 12 are dispersed over linear plasmids and encode primarily HPs.

Thirty-one genes were upregulated by RpoS in strain 297 but not B31 (17 orthologous and 14 non-orthologous; Supplementary Table 4). Ten of the 17 ‘297 only’ orthologous genes also were upregulated in strain B31 but did not meet our stringent cut-off criteria. Three genes (*bb0116/malX-1, bb0578/mcp1*, and *bb0729/gltP*) in the ‘297 only’ orthologous group are dually transcribed by RpoS and RpoD in strain 297 (Caimano et al., 2007; Eggers et al., 2011), which may explain why these genes are missing from strain B31 RpoS DMC regulon. Five of the 14 ‘297 only’ non-orthologous genes (*Bbu297_Z06, _Z19, _Z23, _Z26*, and _*Z27*) are encoded on lp28-6. While the central portion of this linear plasmid is highly similar to lp28-2 in strain B31, the telomeric ends of both plasmids contain non-orthologous genes (Casjens et al., 2012). All five non-orthologous genes on lp28-6 in strain 297 were upregulated only within DMCs (Supplementary Table 4 and Supplementary Figure 6).

#### cp32-Encoded Paralogous Lipoproteins

As with B31, only a small number of *ospE/ospF/elp* genes in strain 297, two *ospF*s (*Bbu297_S41, Bbu297_W43*) and one *elpA1* (*Bbu297_R40*), were RpoS-upregulated in DMCs (Supplementary Figure 7B). Alignment of the upstream sequences for all of the *ospE/ospF/elp* paralogs in 297 and B31 (Supplementary Figure 7A) revealed that the five upregulated by RpoS contain extended -10 regions conferring RpoS promoter selectivity (Eggers et al., 2006). Interestingly, the four RpoS-upregulated OspF paralogs in strains B31 and 297 show substantial sequence divergence (∼30–80% amino acid identity, Supplementary Figure 10), while ElpA1 is unique to 297 (note as well that B31 contains no ElpAs) (Akins et al., 1999). Whereas B31 encodes only one RpoS-upregulated *mlp* (*bbm28/mlpF*, Class I), 297 encodes three, one of which (*Bbu297_R30/Mlp7b*) belongs to Class II (Supplementary Figure 7C). As in B31, both *revA* paralogs in strain 297 are upregulated by RpoS (Table 2). Unlike B31, however, the RevA paralogs in strain 297 are substantially divergent (∼70% identity, Supplementary Figure 7D).

#### Pfam54 Paralogs

As with B31, Pfam54 paralogs in strain 297 can be either positively or negatively regulated by RpoS (Supplementary Figure 8). Closely related paralogs in both strains displayed similar expression profiles; for example, *bba72* (B31) and *Bbu297_a72* were both upregulated by RpoS, while *bba69* (B31) and *Bbu297_a69* were RpoS-repressed (Supplementary Figure 7). However, both strains also harbor strain-specific paralogs that are either up- or down-regulated; *Bbu297_A67a*, a downregulated Pfam54 paralog unique to strain 297 represents one example.

#### RpoS-Repressed Genes

Twenty-two of the 41 genes repressed by RpoS in B31 have identifiable orthologs in strain 297; 16 of these, including *bba15*/*ospA*, *bba62*/*lp6.6* and two *glp* genes (*bb0241/glpK* and *bb0243/glpD*), were repressed by RpoS in both strains (Table 3). *bb0240/glpF* and *bba59*, encoding a surface lipoprotein, were significantly (*q* ≤ 0.05) downregulated in 297 but missed the > 3-fold cut-off. *bba16*/*ospB* and *bba74*, two genes known be to RpoS-repressed in 297 (Caimano et al., 2007; Mulay et al., 2009; Dunham-Ems et al., 2012), are missing from the strain 297 genome fasta file due a confirmed frameshift (*ospB*) or incomplete sequence near the lp54 telomere (*bba74*). Sixteen genes repressed by RpoS in B31, but not 297, are located on plasmids that are either missing (cp9) or highly divergent (lp28-4, lp36, and lp38) in the latter strain (Casjens et al., 2012, 2017). Thirty genes (16 orthologous, 14 non-orthologous) were downregulated in 297 but not B31 (Supplementary Table 5). Seven of the 16 orthologous genes were significantly downregulated in B31 but missed the > 3-fold cut-off.

## Discussion

Alternative σ factors function as master regulators for the transcription of cohorts of genes required for adaptation to adverse conditions or at particular times in the cell cycle (Helmann and Chamberlin, 1988; Wosten, 1998; Feklistov et al., 2014; Campagne et al., 2015). Individual bacteria may encode multiple alternative σ factors, each capable of coordinating a different stress-specific response (Helmann and Chamberlin, 1988; Wosten, 1998; Feklistov et al., 2014; Campagne et al., 2015). Alternative σ factors belonging to the σ^70^ family are grouped according to the presence/absence of four conserved regions (σ_1_ – σ_4_) found in the primary, ‘housekeeping’ σ^70^ factors (Group 1) (Lonetto et al., 1992; Wosten, 1998; Feklistov et al., 2014). Group 2 σ factors, the most closely related to Group 1, contain σ_2_ – σ_4_ and a portion of σ_1_ (σ_1.2_). Groups 1 and 2 σ factors often recognize similar promoters due to their strong structural similarity. Group 3 alternative σ factors contain only σ_2_ – σ_4_ and coordinate a variety of cellular responses, including heat shock (e.g., σ^32^ in *E. coli*), sporulation (e.g., SigF and SigG in *Bacillus subtilis*), and motility (e.g., FliA in *E. coli*). Group 4 σ factors, the most abundant and diverse group, contain only σ_2_ and σ_4_ and are typically associated with extracytoplasmic stress responses (e.g., σ^E^ in *E. coli*). Because of their strict sequence requirements for promoter binding, Group 3 and 4 σ factors generally transcribe limited numbers of genes (Wosten, 1998; Feklistov et al., 2014). In contrast, RpoS (σ^38^), the prototypical Group 2 σ factor in γ-proteobacteria, requires only an extended -10 sequence for binding (Wosten, 1998; Becker and Hengge-Aronis, 2001; Feklistov et al., 2014) and, therefore, can recognize a wide range of promoters. As cells enter stationary phase or following exposure to abiotic stress during exponential growth, accumulation of RpoS promotes the expression of >200 genes involved in the General Stress Response, a survival strategy directed toward the use of alterative carbon and energy sources and the development of multifaceted stress resistance (Weber et al., 2005; Hengge, 2011). Although *B. burgdorferi* RpoS (RpoS_Bb_) shares only 54% amino acid similarity with the *E. coli* RpoS prototype (RpoS_Ec_), herein we show that it retains key features of its proteobacterial namesake, including (i) the presence of region σ_1.2_; (ii) clear structural homology; (iii) promoter recognition *via* an extended -10; and, importantly, (iv) the ability to recognize heterologous RpoS-dependent promoters (Wosten, 1998; Becker and Hengge-Aronis, 2001; Feklistov et al., 2014). Consistent with the ‘generalist’ behavior of Group 2 σ factors (Jorgensen et al., 1999; Weber et al., 2005; Chiang and Schellhorn, 2010; Hengge, 2011), RpoS_Bb_ controls a large suite of genes comprising ∼10% of the spirochete’s genome coding capacity. In contrast to RpoS_Ec_, however, RpoS_Bb_ regulates only a handful of physiological genes, indicating that adaptation to sporadic and diverse environmental and nutritional stress is not the primary function of this alternative σ factor in spirochetes. RpoS_Bb_ has evolved instead to serve as the master regulator of a genetic program for exploiting the window of opportunity for transmission created during tick feeding, and then establishing and sustaining infection in a naïve reservoir host (Hubner et al., 2001; Caimano et al., 2005, 2007; Tilly et al., 2006; Dunham-Ems et al., 2012; Xu et al., 2012). To transcribe *rpoS*_,_ *B. burgdorferi* has appropriated an evolutionarily divergent alternative σ factor, RpoN, that requires an activator complex consisting of Rrp2 and BosR to couple hydrolysis of ATP to formation of an open complex (Yang et al., 2003a; Hyde et al., 2006, 2009; Blevins et al., 2009; Ouyang et al., 2011; Groshong et al., 2012). RpoN-dependent transcription, therefore, links expression of the “effector” σ factor with the environmental signals that initiate and maintain the RpoS-ON state (Ouyang et al., 2008, 2012; Caimano et al., 2016). Our comparison of the strain B31 and 297 RpoS regulons suggests that this invariant regulatory scenario accommodates the genetic diversity that enables *B. burgdorferi* to persist within a wide range of reservoir hosts.

Despite substantial evidence to the contrary (Schwan et al., 1995; Fingerle et al., 1998; Schwan and Piesman, 2000; Belperron and Bockenstedt, 2001; Ohnishi et al., 2001; Mulay et al., 2009; Promnares et al., 2009; Dunham-Ems et al., 2012; Ouyang et al., 2012; Iyer et al., 2015), many investigators continue to believe that OspA and OspC are reciprocally expressed in nymphs during transmission. Using a novel dual reporter system, we found that virtually all spirochetes strongly express *ospA* throughout the transmission blood meal, while downregulation of *ospA* occurs only within the mammal. The reporters also confirmed at a single cell level what has been inferred for years from antibody studies, namely, that repression of *ospA* continues for the duration of infection. Based upon a knock-in genetic strategy conducted with *in vitro-*cultivated organisms, Li, Liang and colleagues (Wang et al., 2013; Shi et al., 2014) posited that BosR blocks transcription of *ospA* by binding to two *cis*-acting sites, one of which overlaps the -10 region. However, the only biologically relevant context for studying the mechanism for repression of *ospA* is the one in which this phenomenon actually occurs — mammalian host adaptation. If BosR alone blocks transcription of *ospA*, then one should see downregulation of OspA in DMC-cultivated Δ*rpoN* and Δ*rpoS* spirochetes, which was not the case. Results presented here and elsewhere (Wang et al., 2013) clearly demonstrate an absolute requirement for an intact RpoN/RpoS pathway for repression of *ospA* in mammals. Using a P*ospA-gfp* transcriptional reporter, we recently mapped the upstream region of *ospA* required for RpoS-mediated repression in DMCs to the gene’s minimal σ^70^ promoter elements (Grove et al., 2017). One potential mechanism for repression involves RpoS-upregulation of a repressor protein that either is not induced or active until spirochetes are within the mammal; RpoS licensing of BosR binding to the *ospA* promoter can be considered a variant of this model. Investigation of BosR as a direct repressor of *ospA* will require a Δ*bosR* strain capable of expressing RpoS at physiological levels in the mammalian host environment. An alternative mechanism, which we favor, is that repression occurs due to increased competition between σ^70^-RNAP and RpoS-RNAP for binding to the *ospA* promoter. According to this ‘σ factor obstruction’ model in mammals, but not ticks, RpoS-RNAP binds to the *ospA* promoter but is unable to form a productive open complex and/or escape to elongation. In support of this idea, Levi-Meyrueis et al. (2015) demonstrated that RpoS-mediated repression did not occur in *Salmonella typhimurium* expressing a *rpoS* point mutant that was able to complex with RNA polymerase but was unable to bind DNA. Why the gatekeeper function of RpoS in *B. burgdorferi* does not occur concomitantly with initiation of the RpoS-ON state during transmission remains an enigma. When RpoS-mediated repression of tick-phase genes was discovered, we postulated that it was induced by mammalian host-specific signals (Caimano et al., 2005, 2007). Subsequently, we noted that Δ*rpoS* organisms in engorged nymphs express significantly higher transcript levels for RpoS-repressed genes than its wild-type parent (Dunham-Ems et al., 2012). This intriguing observation suggests, counterintuitively perhaps, that RpoS-mediated repression is antagonized within feeding ticks and relieved within the mammal. Signaling molecules elicited in spirochetes by the blood meal, most notably c-di-GMP and (p)ppGpp, could be the source of this proposed antagonism (Caimano et al., 2011, 2015; He et al., 2011; Hengge, 2011; Kostick et al., 2011).

Humans, incidental or “dead-end” hosts for *B. burgdorferi*, are at risk for Lyme disease when their activities overlap with habitats in which both competent vectors and reservoirs are present (Radolf et al., 2012; Steere et al., 2016; Stanek and Strle, 2018). Animals are considered to be reservoir competent if they become infected following the bite of an infected tick and can then re-transmit the pathogen to a naïve vector (Keirans et al., 1996; Hanincova et al., 2006; Brunner et al., 2008; Piesman and Schwan, 2010). Because spirochetes are not passed transovarially, infection in a reservoir host must be of long enough duration to serve as a blood meal source for more than one tick life stage (Stewart and Rosa, 2018). To date, efforts to identify genetic determinants of spirochetal persistence in mammals have focused principally upon the Lyme disease spirochete’s many ploys for subversion of innate and adaptive host defenses (Liang et al., 2002a; Weiss and Bockenstedt, 2010; Radolf et al., 2012; Norris, 2014; Marcinkiewicz et al., 2017; Tracy and Baumgarth, 2017). However, bacterial persistence depends on factors besides immune evasion (Lewis, 2010; Gal-Mor, 2019; Gray et al., 2019). To assess the importance of the RpoN/RpoS pathway for persistence, herein, we used a cp9 plasmid-based complementation strategy modeled after that used by Tilly and co-workers for *ospC* (Tilly et al., 2006) to bypass the absolute requirement for RpoS during early infection (Caimano et al., 2005; Dunham-Ems et al., 2012; Xu et al., 2012). Given that the empty vector and *rpoS-*complementing plasmid contain the same origin of replication and differ in size by only ∼1 kb, both should be lost spontaneous at similar rates *in vivo*. With the wild-type control strain, loss of the empty vector had no obvious effect on persistence (i.e., almost all tissues were culture-positive for up to 20 weeks). Loss of the *rpoS*-complementing plasmid, on the other hand, resulted in two separate but inter-related phenotypes. First, RpoS-deficient spirochetes appear to enter a “viable but non-cultivatable” state. Consequently, spirochetes that retained the complementing plasmid were recovered at much higher numbers from tissues. At all time points, however, a small proportion of spirochetes recovered from BbP1754-infected mice lacked the *rpoS*-complementing plasmid, suggesting that plasmid loss did not result in the immediate demise of the bacterium. Second, the presence of low to modest levels of antibodies against OspA and BBA62 in the sera of BbP1754-infected mice is consistent with at least partial loss of RpoS-mediated repression. Because the RpoS DMC regulon includes *mcp4-5*, which encode methyl-chemotaxis proteins (MCPs) that direct spirochetal motility by sensing exogenous chemotactic signals (Charon et al., 2012), admittedly, we cannot rule out the possibility RpoS-deficient spirochetes also have a dissemination defect. However, larvae fed upon mice infected with the *rpoS*-complement strain acquired complemented Δ*rpoS* organisms in proportions roughly similar to those recovered from tissues.

Complementation of Δ*rpoS* spirochetes in these studies represents an interesting counterpoint to complementation of Δ*ospC* organisms in the Tilly studies (Tilly et al., 2006). In the latter, the appearance of OspC antibodies, coinciding with the point in the infectious process (∼2–3 weeks p.i.) when OspC is no longer required (Tilly et al., 2006, 2013; Skare et al., 2016), creates strong pressure against *ospC*-complemented organisms. During early infection, complementation of the Δ*rpoS* mutant is required to drive transcription of *ospC* (Hubner et al., 2001; Dunham-Ems et al., 2012). Once infection has been established, expression of *ospC* in the *rpoS-*complement, as in wild-type spirochetes, is downregulated *via* an upstream *cis*-acting sequence (Xu et al., 2007; Drecktrah et al., 2013), thereby obviating the need to lose the *rpoS*-complementing plasmid. Retention of the *rpoS-*complementing plasmid during late infection conceivably reflects a combination of immune and non-immune pressures on a population-wide basis with the balance weighted toward plasmid retention at any given time. How, then, does RpoS enhance *B. burgdorferi*’s fitness for long-term survival in mammals? As a first step toward identifying persistence genes, we performed RNA-seq on DMC-cultivated strain B31 to re-define the RpoS regulon (Caimano et al., 2007). The identification of *vlsE1* within the B31 RpoS DMC regulon is a noteworthy observation, suggesting that upregulation of this gene occurs in concert with the recombinatorial ‘switching’ that generates extraordinary diversity of VlsE proteins required for evasion of humoral immunity (Zhang et al., 1997; Norris, 2006; Verhey et al., 2018, 2019). We note that two-dimensional gel analysis of skin from *B. burgdorferi*-infected rabbits revealed a remarkable increase in VlsE antigen as infection progressed (Crother et al., 2004). Taking advantage of the Tn library developed by Lin and Norris (Lin et al., 2012), we examined the infectivity of Tn mutants over a longer timeframe (8 weeks) than is typically done in borrelial mutagenesis studies. *B. burgdorferi* requires uptake of peptides *via* the Opp ABC transporter system in mice (Groshong et al., 2017). Of the five oligopeptide-binding proteins in the Opp system, only one, OppA5, is RpoS-upregulated (Caimano et al., 2007; Medrano et al., 2007; Groshong et al., 2017); structural homology modeling using OppA4 as a template suggests that the binding pocket of OppA5 accommodates a unique repertoire of peptide ligands (Groshong et al., 2017). The notion that maintenance of amino acid homeostasis by BBA34 would promote long-term fitness in mammals was borne out by the progressive decrease in recovery of the *bba34* Tn mutant from all tissues examined over time. The increased production of OspA and BBA62 antibodies in mice infected with the *rpoS*-complemented strain revealed another component of the spirochete’s persistence strategy – the gatekeeper function of RpoS. Our data suggest that, once spirochetes are mammalian host adapted, RpoS-mediated repression of tick-phase genes continues for the duration of infection, preventing the production of bactericidal OspA antibodies, while maintaining spirochetes in a state of readiness for re-introduction into the arthropod vector, where they assume the RpoS-OFF state.

The RpoS-ON state encompasses a broad swath of the enzootic cycle and, accordingly, includes genes that enable *B. burgdorferi* to negotiate two vastly different but equally inhospitable milieus. To surmount these environmental challenges, the RpoS regulon undergoes dramatic changes in contour, modulated by transcriptional and post-transcriptional control of *rpoS, trans*-acting factors, regulatory cross-talk from other pathways (ppGpp, c-di-GMP), small RNAs, and differences in promoter strength of individual RpoS-regulated genes (Yang et al., 2005; Lybecker and Samuels, 2007; Medrano et al., 2007; Sanjuan et al., 2009; He et al., 2011, 2014; Miller et al., 2013; Salman-Dilgimen et al., 2013; Sze et al., 2013; Dulebohn et al., 2014; Caimano et al., 2015; Arnold et al., 2018). Although the DMC model is not a perfect facsimile of this complex transcriptional network, it is the only method currently available for identifying genes regulated by RpoS in response to environmental signals encountered within mammals. While many genes are upregulated by RpoS both *in vitro* and within DMCs, the regulons for both B31 and 297 also contained genes that were upregulated only within DMCs. The existence of these ‘DMC only’ upregulated genes provides compelling evidence that RpoS-RNAP holoenzyme has different promoter recognition properties under mammalian host conditions and, along with RpoS-mediated repression, sounds a strong cautionary note regarding the limitations of *in vitro*-cultivated organisms for studying gene regulation in *B. burgdorferi*. With few exceptions, RpoS-upregulated genes studied to date encode lipoproteins, many surface-exposed (Dowdell et al., 2017). The ‘DMC only’ upregulated genes on lp28-2/lp28-6 are unusual because they encode HPs that lack signal peptides and, therefore, function exclusively within the cytoplasm. A number of years ago, Purser and Norris (2000) concluded from short-term needle-inoculation experiments using a small number of mice that lp28-2 is not required for infectivity. The virulence-relatedness of this plasmid needs to be rigorously re-examined with an eye toward persistence.

Our analysis of B31 and 297, two phylogenetically distinct *B. burgdorferi* sensu stricto strains with different origins (tick and human, respectively) (Casjens et al., 2012, 2017), revealed that the RpoS DMC regulon contains two distinguishable components. One consists of a ‘core regulon’ of similarly regulated orthologous genes that presumably provide essential functionalities required to sustain *B. burgdorferi* within its enzootic cycle. While most core genes are chromosomally encoded and highly conserved, *ospC* and *dbpA* are notable exceptions; both of these surface lipoproteins have undergone extensive intra- and inter-species recombination in response to immune and niche-selective forces (Roberts et al., 1998; Barbour and Travinsky, 2010; Mongodin et al., 2013). In humans, OspC types A (B31), K (297), E and I are more often associated with disseminated disease based on clinical symptoms, multiple EM lesions and PCR-positive blood samples (Seinost et al., 1999; Baranton et al., 2001; Jones et al., 2006). The second group includes genes belonging to plasmid-encoded lipoprotein families that have emerged as a result of extensive recombination (Marconi et al., 1996; Stevenson et al., 1998; Akins et al., 1999; Yang et al., 1999; Caimano et al., 2000; Casjens et al., 2000, 2012, 2017; Qiu et al., 2004; Wywial et al., 2009; Brisson et al., 2013; Mongodin et al., 2013). For the cp32-encoded families (*ospF*, *elp*, *mlp*, and *revA*), the subset of genes within each family regulated by RpoS varied between strains. Our transcriptomic data demonstrating that some *ospF*s, one *elp* (*elpA1*) but no *ospE*s are RpoS-upregulated are in line with phylogenetic data arguing that the “Erps” actually originated by insertion of unrelated ancestral protein coding sequences into a single conserved site on different cp32s (Marconi et al., 1996; Akins et al., 1999; Caimano et al., 2000; Brisson et al., 2013). The Pfam54 family is unique in that it contains both RpoS up- and down-regulated genes. The upregulated Pfam54 genes were distributed over four different clades (I, II, III, and V), the members of which are relatively well conserved between strains (Hughes et al., 2008; Wywial et al., 2009). In contrast, all of the RpoS-repressed Pfam54 gene products, including BbCRASP-1, belong to clade IV, which is highly diverse and contains strain-specific members (Wywial et al., 2009). Functional plasticity of these variable plasmid-encoded lipoproteins presumably enables heterogenous spirochetes to coexist in niches with high densities of competent reservoir hosts. Genomic diversification is widely regarded as the driving force behind the dramatic expansion of *B. burgdorferi*’s ecological range during the latter half of the 20th century (Hanincova et al., 2006; Wywial et al., 2009; Barbour and Travinsky, 2010; Brisson et al., 2013; Izac and Marconi, 2019; Tufts et al., 2019). A central theme of contemporary Lyme disease genomics, the extraordinary degree of inter- and intra-species recombination, rearrangements, and decay within the spirochete’s extrachromosomal elements, sets the stage for a new area of investigation – comparative transcriptomics.

## Data Availability

The datasets generated for this study can be found in the NCBI Bioproject, https://www.ncbi.nlm.nih.gov/bioproject/PRJNA546505 and https://www.ncbi.nlm.nih.gov/bioproject/PRJNA547370.

## Ethics Statement

Animal Subjects: This study was carried out in accordance with protocols reviewed and approved by Institutional Animal Care and Use Committees from the UConn Health [Animal Welfare Assurance (AWA) number A347-01], Yale University (AWA number D16-04116), and University of Arkansas for Medical Sciences (AWA number A3063-01) following recommendations in the Guide for the Care and Use of Laboratory Animals of the National Institutes of Health.

## Author Contributions

All authors contributed to the design of the experiments. MC, AG, AB, JM, KH, AL, DG, JS, and JR analyzed the experiments. MC, AG, and JR wrote the manuscript.

## Conflict of Interest Statement

The authors declare that the research was conducted in the absence of any commercial or financial relationships that could be construed as a potential conflict of interest.

## Footnotes

1 http://www.ncbi.nlm.nih.gov/Structure/cdd/cdd.shtml

2 https://www.beiresources.org/
