# Supplementary material for: The RpoS Gatekeeper in Borrelia burgdorferi: An Invariant Regulatory Scheme That Promotes Spirochete Persistence in Reservoir Hosts and Niche Diversity
Source: Front Microbiol. 2019 Aug 21;10:1923. doi: 10.3389/fmicb.2019.01923 (PMC6719511; doi:10.3389/fmicb.2019.01923)
Supplement: Supplementary file 1 [file Data_Sheet_1.pdf]

**Supplemental Table 1. *B. burgdorferi* strains used in this study.**

| Strain | Description | Reference |
|---|---|---|
| BbP1781 | Wild-type B31 5A4 | (Ouyang et al., 2008) |
| EC3/BbP1752 | B31 5A4 Δ*rpoS* mutant | This study |
| EG13/BbP1754 | BbP1752 complemented in *trans* with a wild-type copy of *rpoS* under its native RpoN-dependent promoter (pJSB296) | This study |
| Bb1974 | Wild-type B31 5A4 containing empty vector pJD44 | This study |
| Bb1286 | Wild-type B31 5A4 expressing GFP under the control of the constitutive *flgB* promoter (P*flaB-gfp*) | (Caimano et al., 2015) |
| BbP1981 | Wild-type B31 5A4 expressing GFP under the control of the *ospA* promoter P*ospA-gfp*) and tdTomato under the control of the constitutive *flgB* promoter (P*flgB-tdTomato*) | This study |
| c162 | Wild-type strain 297 clone | (Caimano et al., 2007) |
| c174 | Strain 297 Δ*rpoS* mutant | (Caimano et al., 2007) |
| BbP1572 (BbJSB18-B2) | Strain 297 Δ*rpoN* mutant | (Ouyang et al., 2008) |
| BbP1585 (OY08 A11) | Strain 297 Δ*bosR* mutant | (Ouyang et al., 2011) |
| AG103 | B31 5A18 NP1 clone, transposon mutant parental strain | This study, (Lin et al., 2012) |
| T10TC085 | *bba07* transposon mutant | (Lin et al., 2012) |
| T07TC190 | *bba34* transposon mutant | (Lin et al., 2012) |
| T07TC484 | *bba72* transposon mutant | (Lin et al., 2012) |
| T11TC534 | *bba73* transposon mutant | (Lin et al., 2012) |
| AG135 | *bba34* transposon mutant complemented in *cis* with a wildtype copy of *bba34* under the control of the native promoter | This study |

**Supplemental Table 2. Oligonucleotide primers used in this study**

| Designation | Sequence (5'-3') | Purpose | Reference |
|---|---|---|---|
| 5'rpoS-F1 | GCTCCTTTGTTACGGACTCTTCTGTGTCTTTGC | Inactivation of *rpoS* | This study |
| 3'rpoS-F1AscI | GGCGCGCCCTGAAATTACCCTTGAACAAGATTCAACTC | Inactivation of *rpoS* | This study |
| 5'rpoS-F2AscI | GGCGCGCCGTGAGTAATTAGCTTGTGTTCTCTTACTG | Inactivation of *rpoS* | This study |
| 3'rpoS-F2BssHII | GCGCGCAATTGCATCAGGAATTACACAGCCC | Inactivation of *rpoS* | This study |
| 5'rpoSdiag | GGGACTATTGTCCAGGTTATATCT | Confirmation | This study |
| 3'rpoSdiag | CAGTAAGAGAACACAAGCTAATTACTCACG | Confirmation | This study |
| OspA-BRV2-F | CTCCTTTACTGCTAGCCATAATATATTCTCCTTTTATATT | P*ospA-gf*p reporter | This study |
| OspA-BRV2-R | CGGGACCGGTGCTAGCCCTGAAAGTCCCAAAACTG | P*ospA-gf*p reporter | This study |
| PlessSS-F | ATGAGGGAAGCGGTGATCGCCGA | Confirmation | This study |
| PlessSS-R | TTATTTGCCGACTACCTTGGTGATCTC | Confirmation | This study |
| aph-F-349 | GAAAGCTGCCTGTTCCAAAG | Plasmid retention | This study |
| aph-R-767 | GTCTTCTTCCCAGTTTTCGCAATCCA | Plasmid retention | This study |
| flaB-453-F | AGAGCTTGGAATGCAGCCT | Plasmid retention | This study |
| flaB-993-R | GGGAACTTGATTAGCCTGCG | Plasmid retention | This study |
| PlessGent-F | ATGTTACGCAGCAGCAACGATG | Confirmation | This study |
| PlessGent-R | TTAGGTGGCGGTACTTGGGTCCA | Confirmation | This study |
| 5'bb0418tndiag | ATGTTAATAAAAAAAATTTGCTTTTGTTTG | Confirmation | This study |
| 3'bb0418tndiag | TTAGTATTTATAAGTTATAGACATTCCAATAGAATCGTAA | Confirmation | This study |
| 5'bba04tndiag | GCTTCCATCAACAGGAGAAACAAGATAAGAATAC | Confirmation | This study |
| 3'bba04tndiag | CGCATGTTAAACAGCTTGATAAAAGAGATTAGC | Confirmation | This study |
| 5'bba07tndiag | AGAGCCATTTTAGCCTTTCTTT | Confirmation | This study |
| 3'bba07tndiag | TAAACGCTGTTTTTGTTCTTCAATGTTTTCTAT | Confirmation | This study |
| 5'bba33tndiag | ATGTCTTTTAAGTTGTAGTTCT | Confirmation | This study |
| 3'bba33tndiag | GTCAATGCTGTTACTAAGAATG | Confirmation | This study |
| 5'bba34tndiag | TTATTCTTCTATAGGTTTTATTTCTGATAGGGCAAATCTT | Confirmation | This study |
| 3'bba34tndiag | ATGATAATAAAAAAAAGAGGACTTTTAATACTGGGCATTG | Confirmation | This study |
| 5'bba72tndiag | GCATTAGGTCAAATTCTGCGTGTATTAGTAGATCG | Confirmation | This study |
| 3'bba72tndiag | GTAGTGTATGTGGTCACAACAGGTTTTTAGCGG | Confirmation | This study |
| 5'bba73tndiag | CATTTTTAAAGAACTGGCTTTG | Confirmation | This study |
| 3'bba73tndiag | CCTTGTTTGCACCCTCAGCAAC | Confirmation | This study |
| 5'bbb09tndiag | GCATGAATGCCGGTTTTAAATTTACCATCTCC | Confirmation | This study |
| 5'bbb09tndiag | GATAATGCTTTTATTAAAGCTAGATTTTACTTTGAGTTCTGC | Confirmation | This study |
| 5'bba34c-F1 | *CGGTACCCGGGGATCC*GCATTTTACAGGTTTTTGAACACT CTCATC | Cloning | This study |
| 3'bba34c-F1 | *CTTCCTTGAAGCTCG*CCCTATTTGTGAAAAAGTTTAAAAAT CAGTTATTC | Cloning | This study |
| 5'bba34c-Strep | *TTTTCACAAATAGGG*CGAGCTTCAAGGAAGATTTCCTATTA AGGTTGAAC | Cloning | This study |
| 3'bba34c-Strep | *GGGTCAAAATTTTGA*CGGCGACGTCATTATTTGCCGACTA CCTTGGTGA | Cloning | This study |
| 5'bba34c-F2 | *ATAATGACGTCGCCG*TCAAAATTTTGACCCTATTTATGAAA ACTTATTTT | Cloning | This study |
| 3'bba34c-F2 | *CGACTCTAGAGGATCC*CAATCTATTCAAATGTATTTCTGGG GGAGT | Cloning | This study |
| bba34-F | CAAGCGATGTTGGTTCGTTTC | qRT-PCR | (Iyer et al., 2015) |
| bba34-R | TACTGGGCATTGCTACTGTAATC | qRT-PCR | (Iyer et al., 2015) |

Underline indicate restriction enzyme sites.
Italics indicates overlap for In-Fusion cloning

**Supplemental Table 3. RpoS-deficient organisms are avirulent in C3H mice by needle-inoculation and tick transmission.**

| | Syringe-inoculated mice[1] | | | | | | Tick-infected mice[2] | |
|---|---|---|---|---|---|---|---|---|
| | Ear | skin | Lymph nodes | Joints | Bladder | Heart | Serology[3] | Ear |
| WT | 6/6 | 6/6 | 6/6 | 6/6 | 6/6 | 6/6 | 5/5 | 5/5 |
| Δ*rpoS* | 0/6 | 0/6 | 0/6 | 0/6 | 0/6 | 0/6 | 0/5 | 0/5 |
| *rpoS*comp | 6/6 | 6/6 | 6/6 | 6/6 | 6/6 | 4/6 | 5/5 | 4/5 |

[1]Tissues were collected from syringe-inoculated mice two weeks post-inoculation with $10^5$ organisms. Data represent two independent experiments, 3 mice per strain, per experiment.

[2]Ear tissues were collected from C3H/HeJ mice 2 weeks post-repletion with ~10-15 nymphs per mouse.

[3]Serology was performed using serum collected from tick-infected C3H/HeJ mice 2 weeks post-repletion immunoblotted against whole cell lysates of wild-type strain B31 5A4.

**Supplemental Table 4. *B. burgdorferi* genes upregulated in DMCs by RpoS in strain 297 but not strain B31.**

| Gene ID | Genomic element[1] | Gene | Description | Lipo[2] | Fold-Regulation[3] | Closest match in B31[4] | B31 Fold-Reg in DMCs[5] |
|---|---|---|---|---|---|---|---|
| Bbu297_Z26 | lp28-6 | . | Hypothetical protein | ND | 98.25 | . | |
| Bbu297_A65 | lp54 | . | Lipoprotein putative (Pfam54) | Surface | 79.24 | BBA65 | 3.29 (6) |
| Bbu297_Z23 | lp28-6 | . | Lipoprotein putative | ND | 63.37 | . | |
| Bbu297_A71 | lp54 | . | Hypothetical protein | ND | 35.38 | . | . |
| Bbu297_Z06 | lp28-6 | . | Hypothetical protein | ND | 14.83 | . | . |
| Bbu297_Z27 | lp28-6 | . | Hypothetical protein | ND | 14.12 | . | . |
| Bbu297_A04 | lp54 | . | Putative antigen S2 truncated | Surface | 14.10 | BBA04 | 2.21 |
| Bbu297_E04 | lp25 | . | outer membrane protein | Surface | 8.51 | BBI42, BBK53 | ND (7) |
| Bbu297_Z19 | lp28-6 | . | Hypothetical protein | ND | 7.53 | . | . |
| Bbu297_J18 | lp36 | . | ABC transporter ATP-binding protein-like protein | . | 7.31 | BBJ26 | 10.24 (6) |
| Bbu297_R41 | cp32 | *2.9-11* | Truncated Mlp | ND | 6.13 | . | . |
| Bbu297_J20 | lp38 | . | Hypothetical protein | . | 5.82 | BBJ24 | 9.21 (6) |
| Bbu297_I24 | lp28-4 | . | Outer membrane protein P13 | ND | 5.68 | . | . |
| BB0842 | main | *arcB* | Ornithine carbamoyltransferase catabolic | . | 5.63 | BB0842 | 3.07 (6) |
| Bbu297_R40 | cp32 | *elpA1* | ElpA1 (Erp45) | ND | 5.63 | . | . |
| Bbu297_K32 | lp38 | . | Lipoprotein putative | Surface | 4.89 | BBI42, BBK53 | ND (7) |
| Bbu297_K01 | lp38 | . | Hypothetical protein | | 4.84 | . | . |
| Bbu297_A73 | lp54 | . | P35 antigen putative lipoprotein (Pfam54) | Surface | 4.71 | BBA64 | -3.63 |
| BB0689 | main | . | Hypothetical protein | Surface | 4.40 | BB0689 | 3.78 (6) |
| Bbu297_J17 | lp36 | . | Permease putative domain protein | . | 4.26 | BBJ27 | 5.95 (6) |
| BB0400 | main | . | Hypothetical protein | . | 3.89 | BB0400 | 4.38 (6) |
| BB0040 | main | *cheR-1* | Chemotaxis protein methyltransferase | . | 3.78 | BB0040 | 3.3 (6) |
| BB0116 | main | *malX-1* | PTS system maltose and glucose-specific IIABC component | . | 3.73 (8) | BB0116 | 5.22 (6) |
| Bbu297_W45 | cp32 | . | TM2 domain family | ND | 3.72 | . | . |
| Bbu297_S02 | cp32 | . | DUF1357 SF | ND | 3.49 | . | . |
| BB0729 | main | *gltP* | Glutamate transporter | . | 3.48 (8) | BB0729 | 2.71 |
| Bbu297_S03 | cp32 | . | Lyme disease proteins of unknown function | ND | 3.23 | . | . |
| Bbu297_S07 | cp32 | . | BBM07-like protein | ND | 3.14 | . | . |
| BB0147 | main | *flaB* | Flagellin | . | 3.11 | BB0147 | 1.35 |
| BB0843 | main | . | Arginine-ornithine antiporter | . | 3.09 | BB0843 | 2.31 |
| BB0578 | main | *mcp-1* | Methyl-accepting chemotaxis protein | . | 3.07 (8) | BB0578 | 3.47 (6) |

[1] Corresponding genome location for respective genes in strain 297. lp28-6 in 297 and lp28-2 in strain B21 share a large number of orthologous genes and likely represent divergent forms of an ancestral plasmid. Because lp25 was missing from the clonal 297 isolate used for whole genome sequencing, the corresponding plasmid from the very closely-related strain JD-1 (Casjens et al., 2012) was used for mapping.

[2] Lipoprotein localization for strain B31 orthologs based on (Zuckert et al., 2004) and/or previously published reports. ND, not determined.

[3] Values are for the wildtype vs Δ*rpoS* mutant comparison. Highlighting is used to indicate genes that were upregulated at least 3-fold with adjusted p value (q) <0.05.

[4] Closest matches in strain B31 based on pairwise BLAST-P. Proteins sharing >90% identity and located on similar genetic elements were considered orthologous. Dots (·) indicates genes for which no clear ortholog could be identified in strain B31.

[5] Values are for the B31 wildtype vs Δ*rpoS* mutant comparison.

[6] Significant in strain B31 DMC wildtype vs Δ*rpoS* mutant but not significant (<3-fold regulation and/or *q*>0.05) in the *rpoS*comp vs Δ*rpoS* mutant comparison.

[7] Closest match could not be determined based on amino acid sequence identify. Shares 89% amino acid identity with BBI42 (4.27-fold) and BBK53 (3.22-fold) in strain B31.

[8] Regulated by both RpoS and RpoD (dually-transcribed) (Caimano et al., 2007).

**Supplemental Table 5. *B. burgdorferi* genes repressed by RpoS in DMCs in strain 297 but not strain B31.**

| Gene ID | Genomic element[1] | Gene | Description | Lipo[2] | 297 Fold-Reg in DMCs[3] | Closest match in B31[4] | B31 Fold-Reg in DMCs[5] |
|---|---|---|---|---|---|---|---|
| Bbu297_A67a | lp54 | . | Antigen P35 homolog (Pfam54) | ND | -16.67 | . | . |
| Bbu297_K24 | lp38 | . | Hypothetical protein | . | -10.49 | BBK34 | -1.66 |
| Bbu297_V30 | cp32 | . | Hypothetical protein | ND | -8.28 | . | . |
| Bbu297_A41 | lp54 | . | Hypothetical protein | . | -6.31 | BBA41 | -10.43 (6) |
| Bbu297_A53 | lp54 | . | Bbs27 protein | . | -5.69 | BBA53 | -6.03 (6) |
| Bbu297_A38 | lp54 | . | Conserved hypothetical protein | . | -5.16 | . | . |
| Bbu297_A40 | lp54 | . | Lyme disease protein | . | -5.04 | BBA40 | -11.2 (7) |
| Bbu297_A39 | lp54 | . | Hypothetical protein | ND | -5.02 | . | . |
| Bbu297_K27 | lp38 | . | Transposase-like protein | ND | -4.74 | . | . |
| BB0542 | main | . | Hypothetical protein | P-IM | -4.45 | BB0542 | 1.22 |
| Bbu297_J02 | lp36 | . | Hypothetical protein | ND | -4.43 | . | . |
| Bbu297_A42 | lp54 | . | Hypothetical protein | . | -4.16 | BBA42 | -5.79 (6) |
| Bbu297_A45 | lp54 | . | Hypothetical protein | . | -4.06 | BBA45 | -3.61 (7) |
| Bbu297_A54 | lp54 | . | Hypothetical protein | . | -3.84 | BBA54 | -6.74 (6) |
| BBUJD1_E06 | lp26 | *bptA* | Borrelia persistence in ticks protein A | . | -3.82 | BBE16 | -2.76 |
| BBUJD1_E07 | lp25 | . | Conserved hypothetical protein | ND | -3.68 | . | . |
| Bbu297_A43 | lp54 | . | Hypothetical protein | . | -3.63 | BBA43 | -3.92 (6) |
| Bbu297_B13 | cp26 | *pf49* | PF-49 protein | ND | -3.56 | . | . |
| Bbu297_J22 | lp36 | . | Borrelia ORF-A SF | . | -3.54 | BBJ19 | -2.08 |
| Bbu297_J26 | lp36 | . | Transposase-like protein | ND | -3.40 | . | . |
| Bbu297_H08 | lp28-4 | . | RepU | . | -3.37 | BBH13 | -2.92 |
| Bbu297_J06 | lp36 | . | Virulent strain associated lipoprotein | ND | -3.29 | . | . |
| Bbu297_B06 | cp26 | *chbB* | PTS system Chitobiose-specific IIB protein | . | -3.27 | BBB06 | -1.57 |
| Bbu297_B07 | cp26 | . | outer surface protein-like protein | . | -3.18 | BBB07 | -1.42 |
| Bbu297_H23 | lp28-4 | *pf49* | PF-49 protein | ND | -3.05 | . | . |
| Bbu297_P41 | cp32 | . | Conserved hypothetical protein | ND | -3.03 | . | . |
| Bbu297_V41 | cp32 | . | Conserved hypothetical protein | ND | -3.03 | . | . |
| Bbu297_X41 | cp32 | . | Conserved hypothetical protein | ND | -3.03 | . | . |
| Bbu297_B27 | cp26 | . | Lipoprotein putative | P-IM | -3.01 | BBB27 | -1.58 |
| Bbu297_J25 | lp36 | *pf49* | PF-49 protein | . | -3.00 | BBJ16 | 1.7 |

[1] Corresponding genome location for respective genes in strains B31 and 297.

[2] Lipoprotein localization for strain B31 orthologs based on (Zuckert et al., 2004) and/or previously published reports. P_IM, periplasmic leaflet of inner membrane. ND, not determined.

[3] Values are for the wildtype vs Δ*rpoS* mutant comparison. Highlighting is used to indicate genes that were upregulated at least 3-fold with adjusted *p* value (*q*) <0.05.

[4] Closest matches in strain B31 based on pairwise BLAST-P. Proteins sharing >90% identity and located on similar genetic elements were considered orthologous. Dots (·) indicates genes for which no clear ortholog could be identified in strain B31.

[5] Values are for the B31 DMC wildtype vs. Δ*rpoS* mutant comparison.

[6] Not significant (*q*>0.05).

[7] Significant in strain B31 DMC wildtype vs. Δ*rpoS* mutant but not *rpoS*comp vs Δ*rpoS* mutant comparison.

A

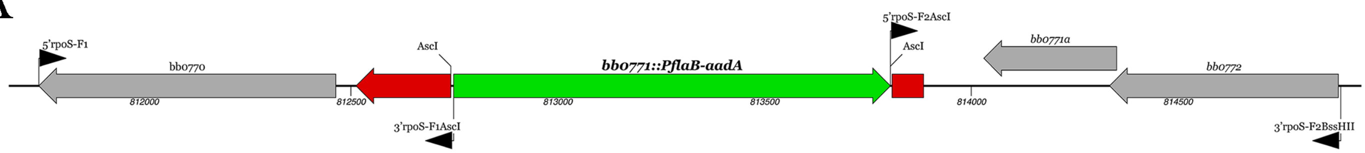


B

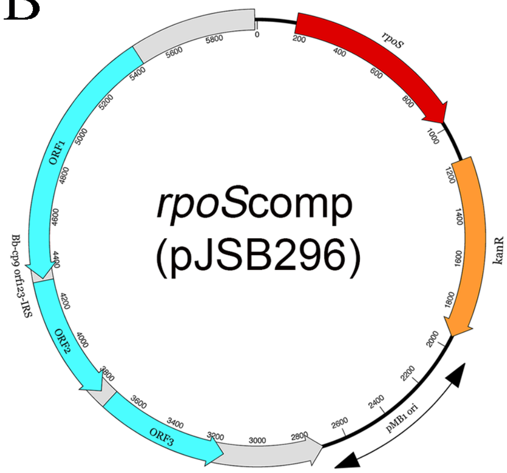


**Supplemental Figure 1. Generation of strain B31 5A4 Δ*rpoS* mutant and *trans*-complemented strains.** **A**. Cartoon depicting the chromosomal insertion event using pJSB634A to generate a strain B31 5A4 Δ*rpoS* mutant. **B**. Plasmid map for pJSB296, the cp9-based shuttle vector used for *trans* complementation of the Δ*rpoS* mutant.

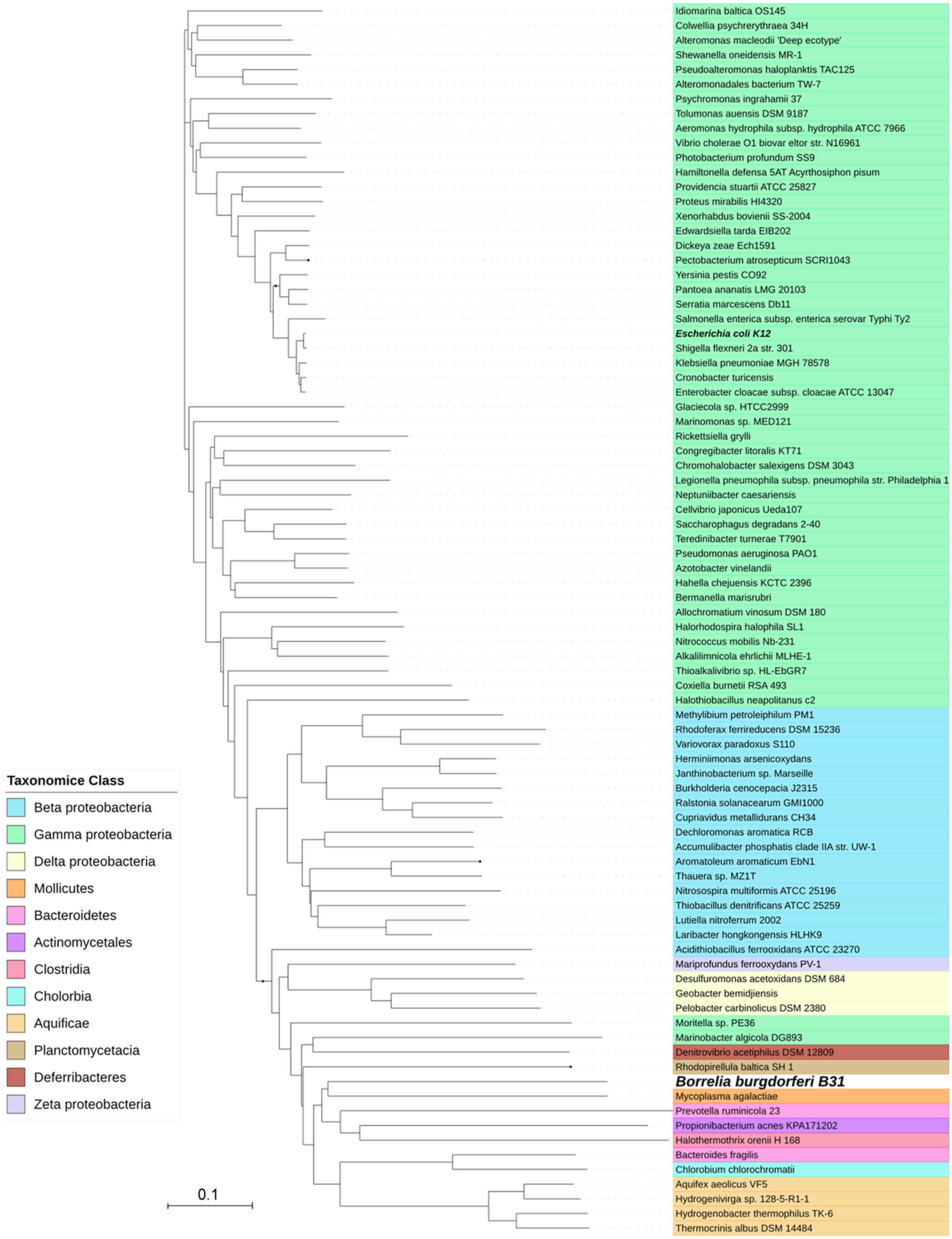


**Supplemental Figure 2. Phylogenetic analysis of RpoS homologs**. Annotated RpoS homologs available in the SEED database (Disz et al., 2010) were aligned using Omega (Sievers et al., 2011) with default settings. Unrooted neighbor-joining trees were visualized and annotated using Interactive Tree of Life (iTOL, v 4.3) (Letunic and Bork, 2016).

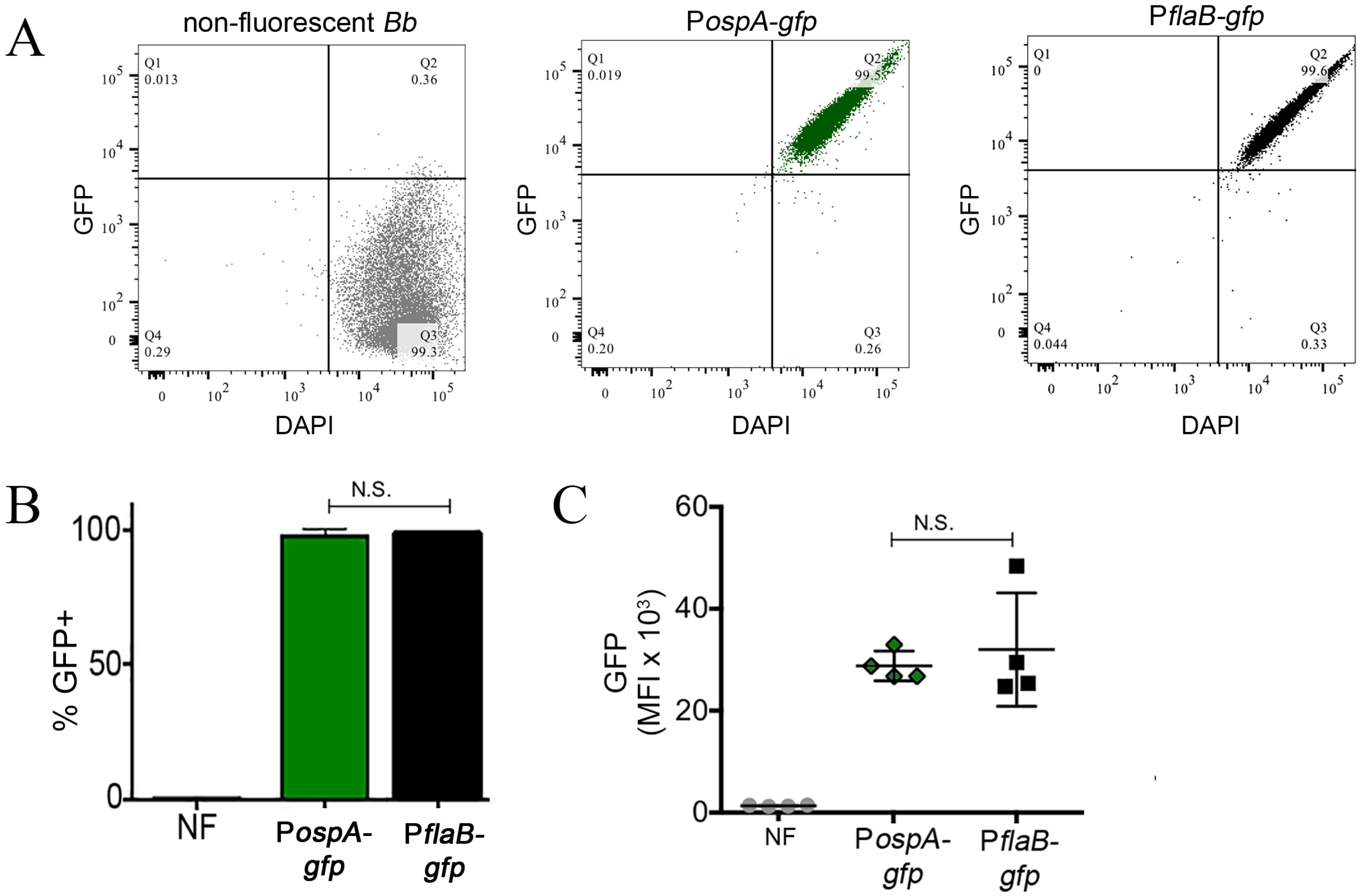


**Supplemental Figure 3. Comparison of GFP fluorescence in P*ospA-gfp/PflgB-tdTomato* (dual color) and P*flaB-gfp* (GFP only) strains**. **A**. Representative quad plots for the non-fluorescent (NF) control (BbP1781), P*ospA-gfp* (BbP1981), and P*flaB-gfp* (BbP1286) stained with DAPI. **B.** Bar graph comparing the percentage (%) of GFP+ NF control, P*ospA-gfp* and P*flaB-gfp* subpopulations. **C**. Relative GFP mean fluorescence intensities (MFIs) of NF control, P*ospA-gfp* and P*flaB-gfp* DAPI+ events. Data in panels B and C represent the averages and standard errors of the mean of four biologically-independent cultures for each strain. Significance was determined using a Mann-Whitney test with $p \leq 0.05$ being considered significant. N.S., Not significant.

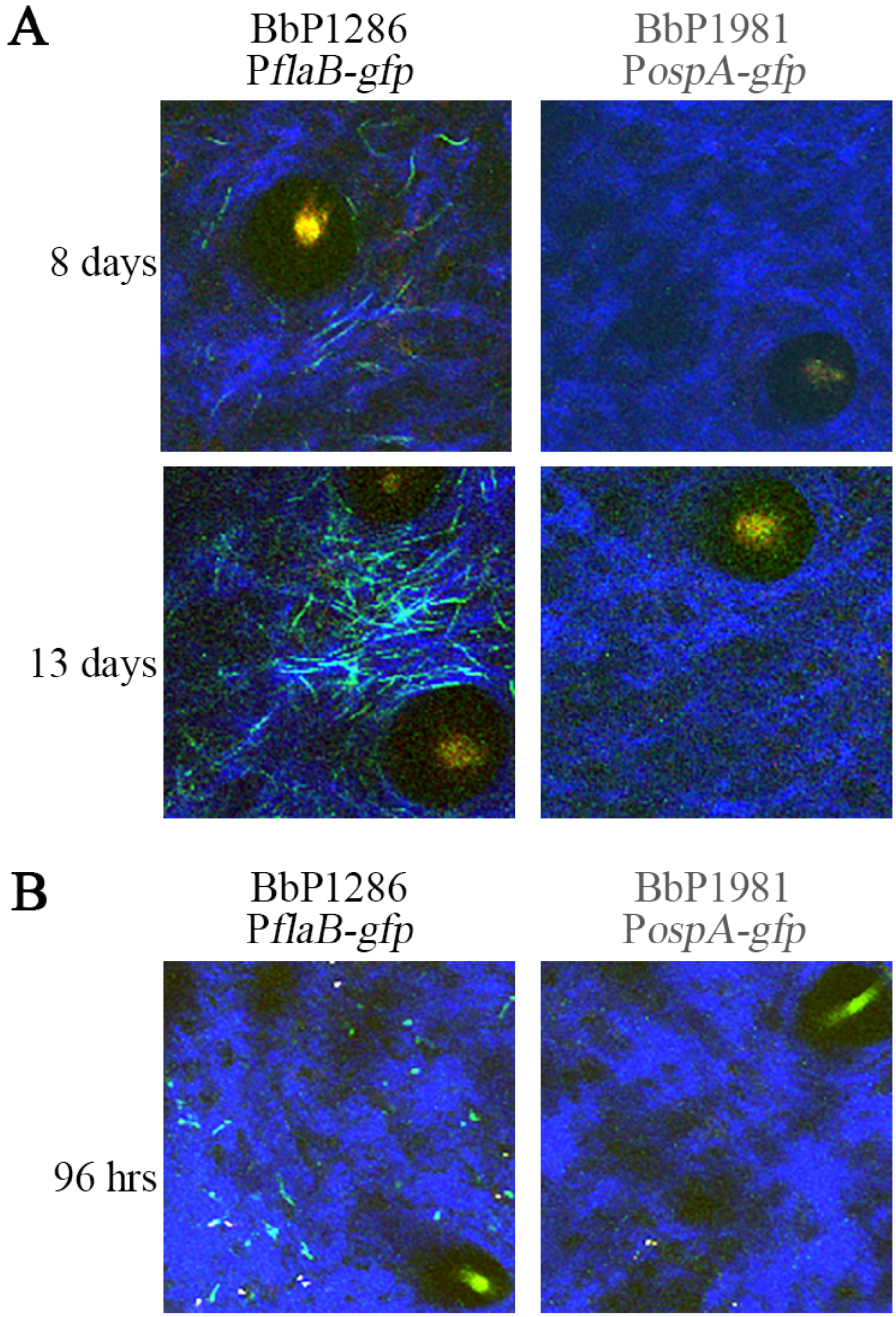


**Supplemental Figure 4. Expression of *ospA* is repressed in mice following needle-inoculation and is not turned on in tissue surrounding the bite site during acquisition. A.** Representative composite two-photon microscopy images of ears from *Myd88*$^{-/-}$ mice either 8 or 13 days after needle-inoculation with *B. burgdorferi* expressing a P*flaB-gfp* (BbP1286) or P*ospA-gfp* (BbP1981) fluorescent reporter. **B**. Representative composite two-photon microscopy images of tissue surrounding the bite site during acquisition on *Myd88*$^{-/-}$ mice infected with *B. burgdorferi* expressing a P*flaB-gfp* (BbP1286) or P*ospA-gfp* (BbP1981) fluorescent reporter. Images were acquired ~96 hours post-placement of naïve nymphs. Three-dimensional z-stack images were rendered using Volocity software from images of sequential *x,y* planes taken at different levels. Hair follicles and dermal collagen fibers fluoresce yellow-orange and blue, respectively, due to second-harmonic generation. A minimum of 20 fields per tissues were examined.

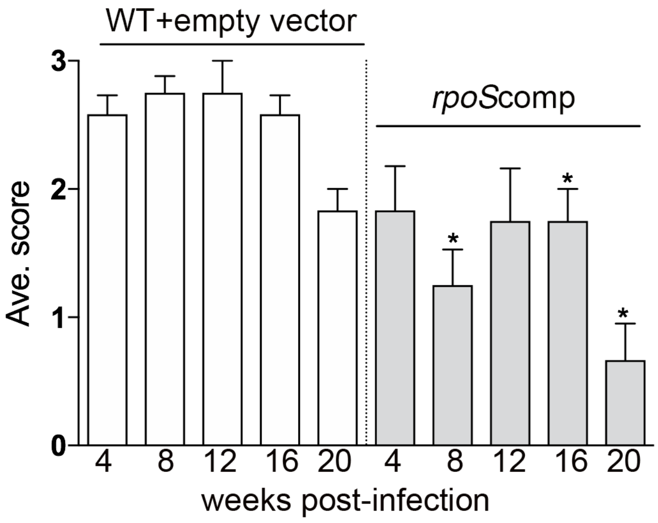


**Supplemental Figure 5. RpoS is required for persistence in murine tissues.** Bar graphs depicting the average culture scores for tissues collected from mice infected with WT+empty vector (BbP1974) and *rpoS*comp (BbP1754) strains (3 mice per group, per strain, per time point). Scores are based on culturing data for individual tissues presented in Table 1. +++, ++, + and negative (-) culture data points were assigned scores of 3, 2, 1 and 0, respectively. Numbers on *x*-axis indicate weeks post-infection. *p* values for pairwise comparisons (WT+empty vector and *rpoS*comp at the same time point) were determined using a two-tailed *t* test. *, $p \leq 0.05$. This figure is a graphical representation of data presented in Table 1.

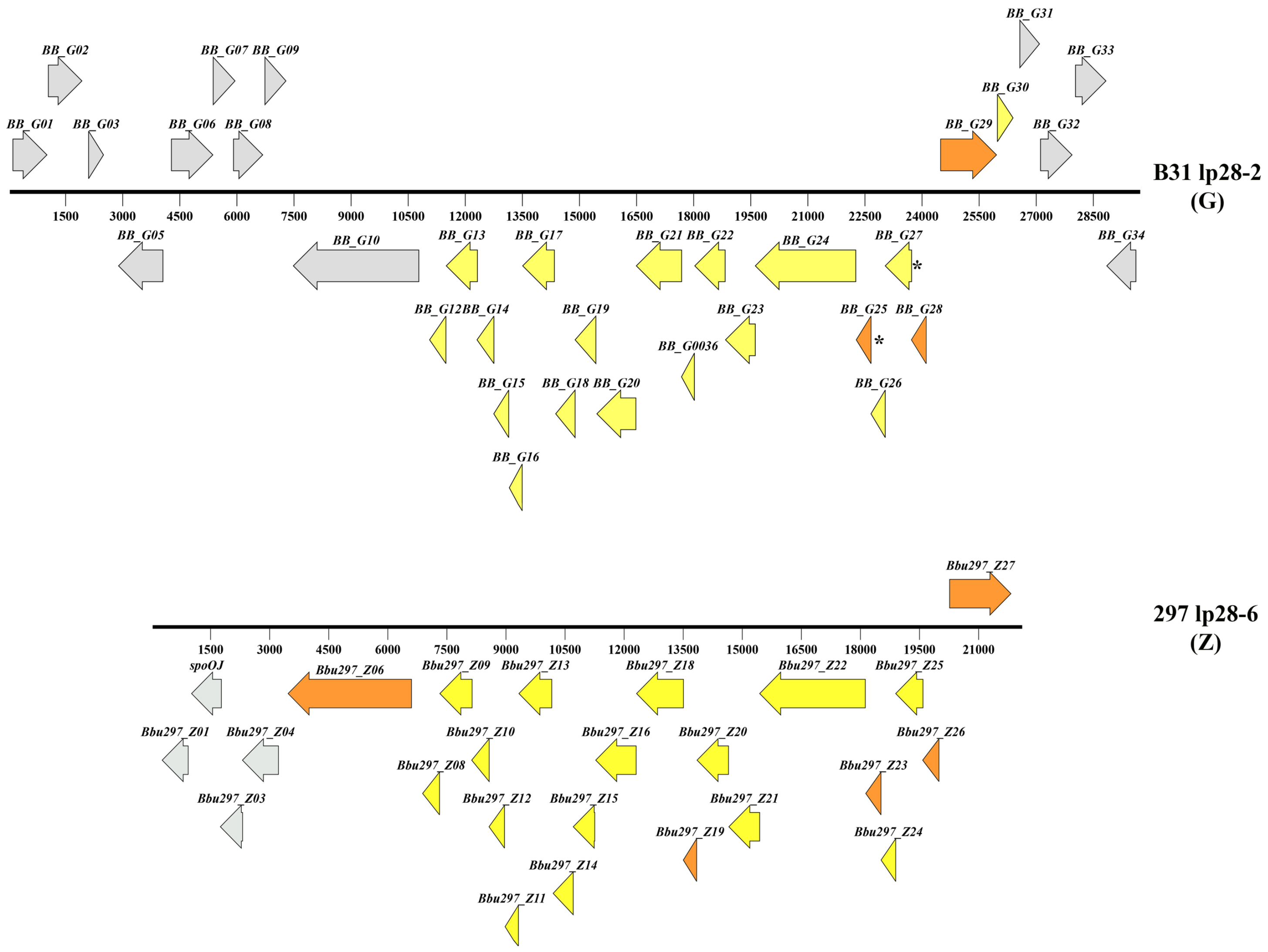


**Supplemental Figure 6. RpoS upregulates a subset of genes encoded on lp28-2 in B31 and the orthologous plasmid in strain 297 (lp28-6) only within DMCs.** Orthologous and non-orthologous RpoS-upregulated genes in strains B31 and 297 are shown in yellow and orange, respectively. Genes in grey were expressed but not regulated by RpoS. *, *bbg25* and *bbg27* were upregulated by RpoS *in vitro* following temperature-shift as well as in DMCs.

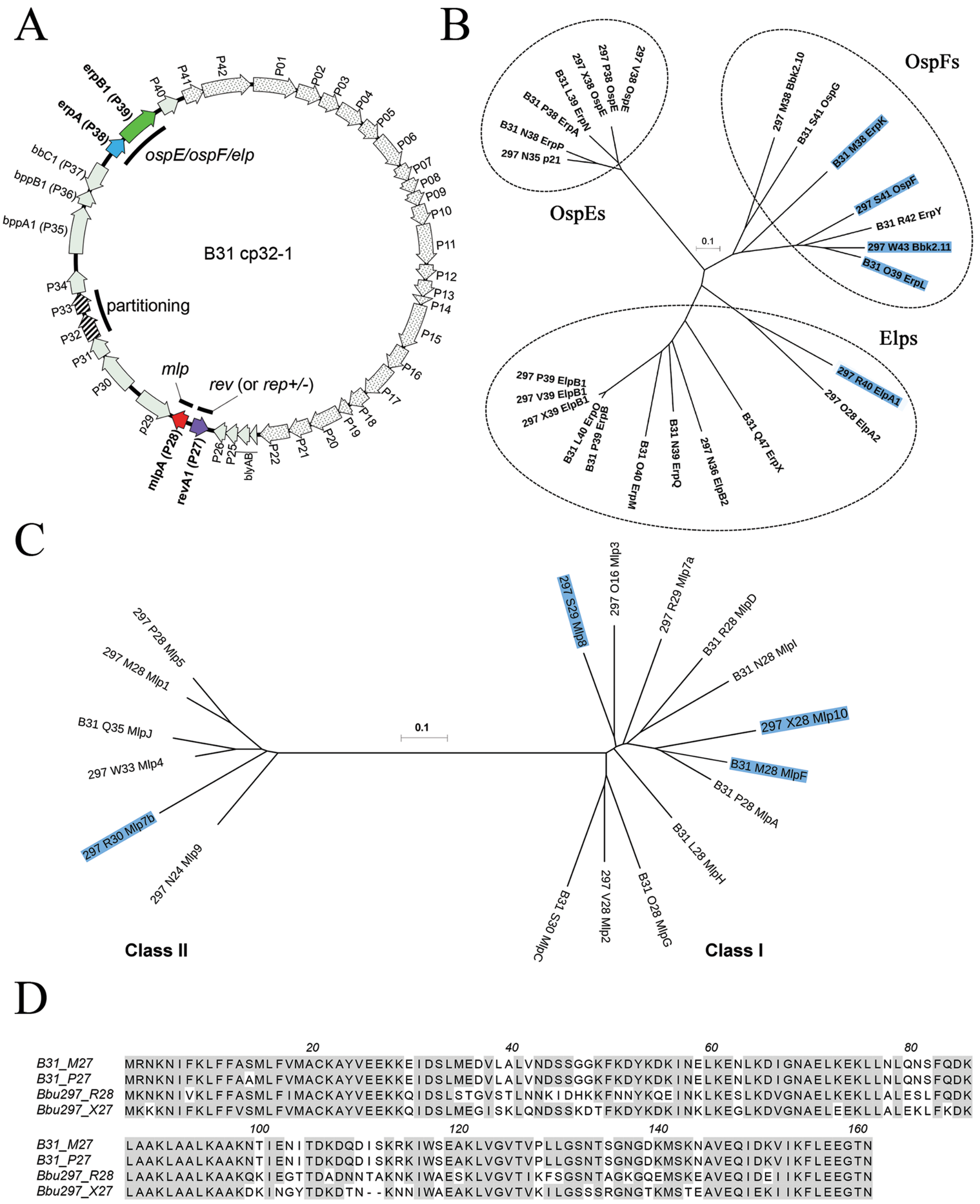


**Supplemental Figure 7**. **RpoS-upregulates expression of a subset of cp32-encoded variable lipoproteins belonging to the *ospE/ospF/elp*, *mlp*, and *revA* paralogous gene families**. **A**. Representative cp32 plasmid showing the locations of *ospE/ospF/elp*, *mlp* and *rev* paralogous lipoprotein genes and the plasmid-specific partitioning region. In strains B31 and 297, only two cp32 plasmids (cp32-1 and cp32-6 in B31 and cp32-1 and cp32-2 in 297) contain *rev* loci; all other cp32s contain overlapping *rep+/-* loci (Casjens et al., 1997;Yang et al., 1999;Caimano et al., 2000). Phylogenetic analyses of OspE/OspF/Elp (**B**) and Mlp (**C**) full-length lipoproteins from strains B31 and 297 generated in Clustal Omega (Sievers et al., 2011) using default settings. Unrooted Neighbor-joining trees were visualized and annotated using Interactive Tree of Life (iTOL, v 4.3) (Letunic and Bork, 2016). Blue shading indicates cp32-encoded paralogs upregulated by RpoS in either strain. RpoS-upregulated paralogs in panels B and C are shaded blue. **D**. Multiple sequence alignment of RpoS-upregulated RevA paralogs from strains B31 and 297.

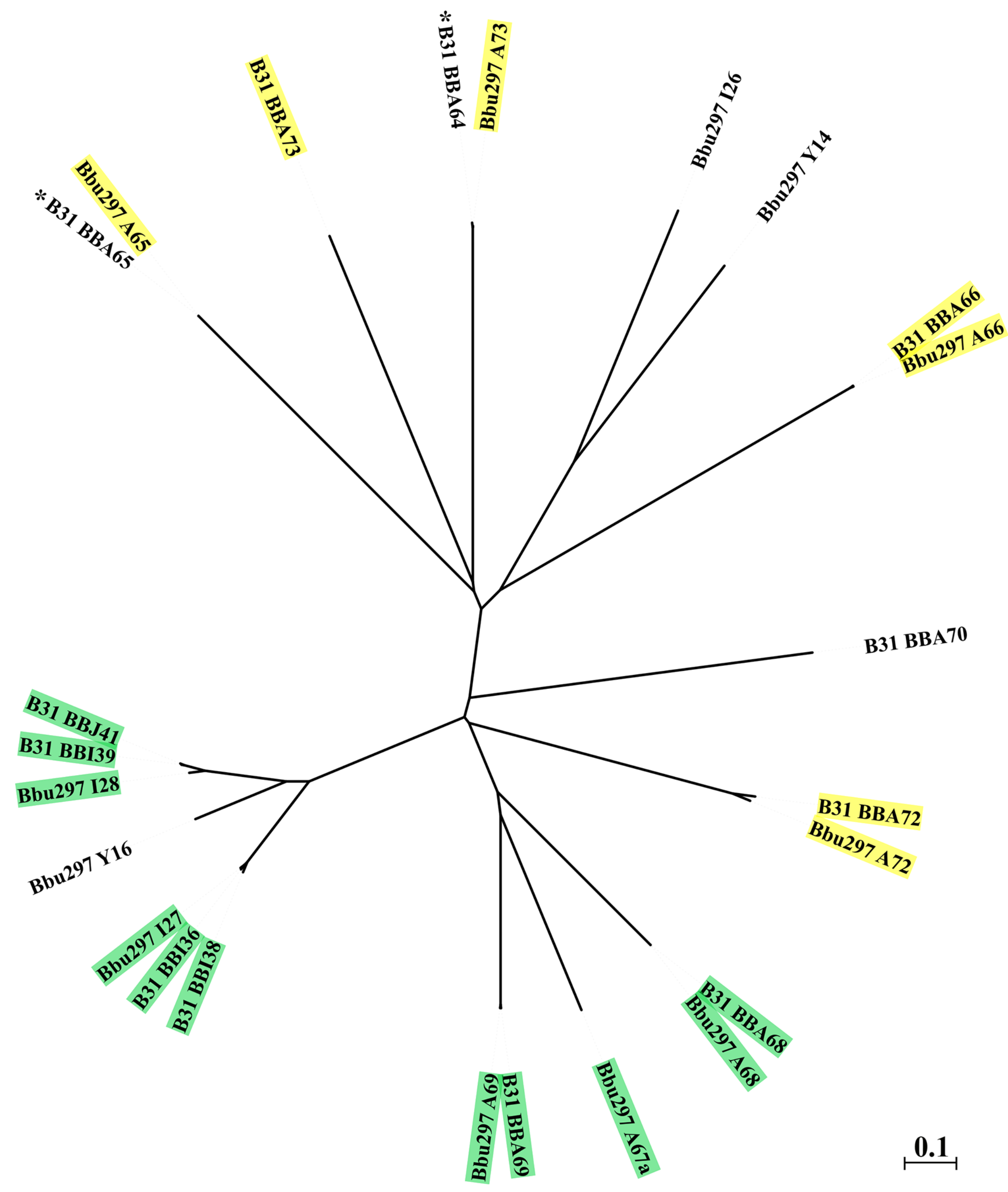


**Supplemental Figure 8. RpoS-regulated Pfam54 paralogs in strains B31 and 297.** Phylogenetic analysis of Pfam54 proteins from strains B31 and 297 was generated in Clustal Omega (Sievers et al., 2011) using default settings. Unrooted Neighbor-joining trees were visualized and annotated using Interactive Tree of Life (iTOL, v 4.3)(Letunic and Bork, 2016). Shading indicates Pfam54 paralogs upregulated (yellow) or repressed (green) by RpoS in the designated strain. Asterisks (*) are used to indicate Pfam54 genes that appear to be RpoS-regulated but were exclude for the following reasons: *bba64* was significantly upregulated by RpoS *in vitro* (11.79-fold, $q \leq 0.05$) but not in DMCs. Of note, RNA-seq data for *Bbu297_a73*, the strain 297 *bba64* ortholog, agree with previous microarray data using DMC-cultivated strain 297 (Caimano et al., 2007). *bba65* was significantly upregulated (3.29-fold, $q \leq 0.05$) in the DMC wildtype vs. Δ*rpoS* mutant comparison but not the *rpoS*comp vs. Δ*rpoS* mutant comparison (2.55-fold).

A

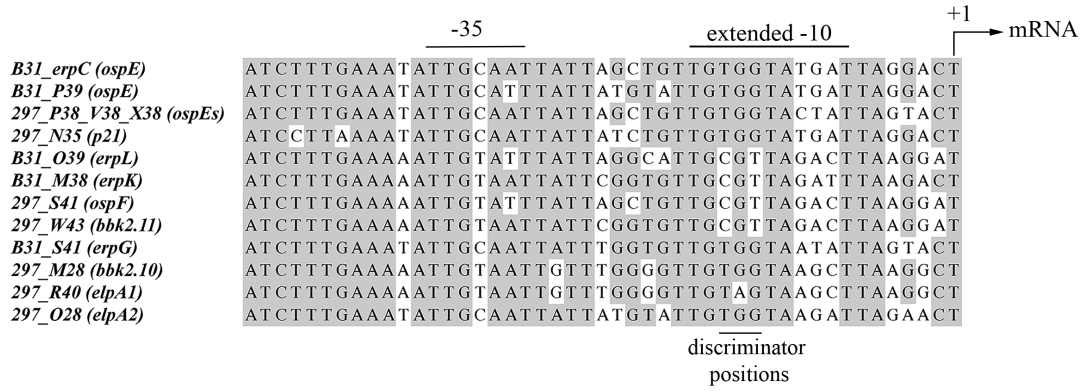


B

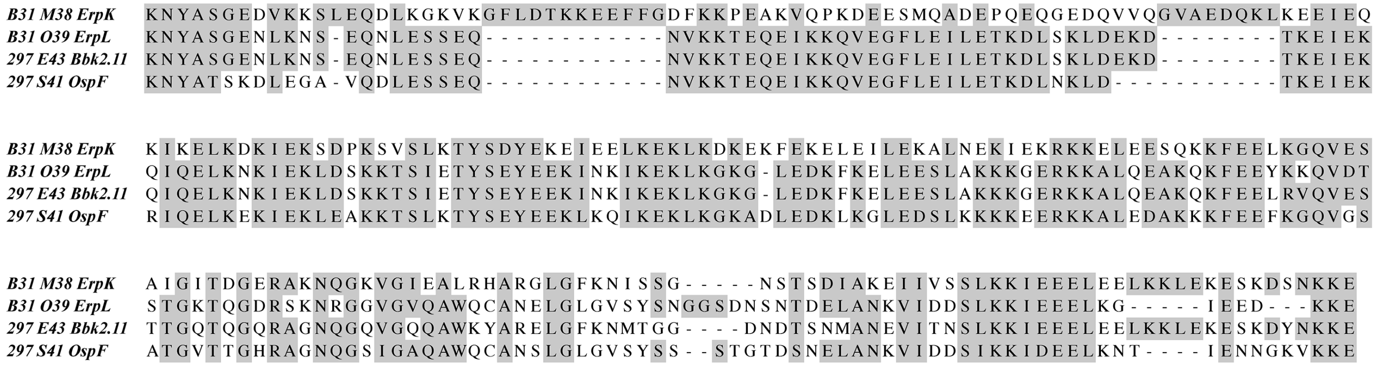


**Supplemental Figure 9**. Only a subset of *ospE/ospF/elp* paralogs are regulated by RpoS in DMCs. **A**. The upstream regions for RpoS-upregulated *ospF* and *elpA* paralogs contain polymorphisms known or predicted to be involve in promoter selectivity by RpoS in *B. burgdorferi* strains B31 and 297 (Eggers et al., 2004;2006). **B**. Multiple sequence alignment of RpoS-upregulated OspF paralogs in strains B31 and 297 show substantial divergence at the amino acid level.

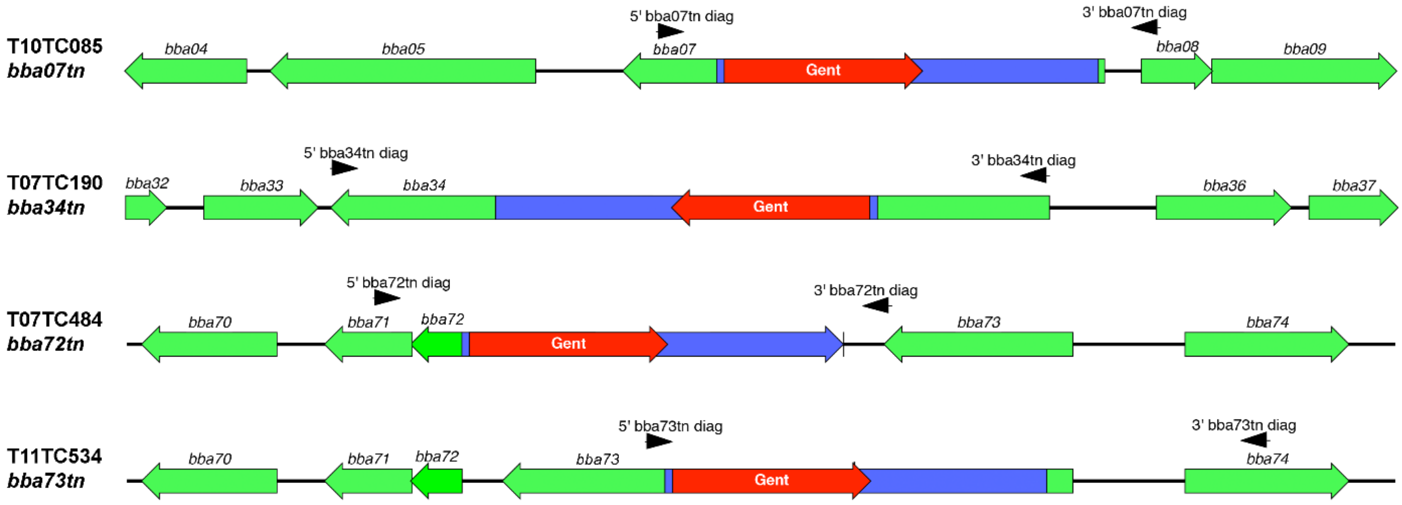


**Supplemental Figure 10**. Schematic depiction of genome location and insertion sites for strain B31 *bba07*, *bba34*, *bba72* and *bba73* transposon (Tn) mutants used in this study. Numbers on the left indicate the unique designation given to each mutant by Lin *et al*. (Lin et al., 2012) Primers used to confirm the insertion sites for each mutation are indicated by black arrows. The gentamicin-resistance cassette (Gent) and signature-tagged *himar1* transposon are shown in red and blue, respectively.

## REFERENCES


Caimano, M.J., Dunham-Ems, S., Allard, A.M., Cassera, M.B., Kenedy, M., and Radolf, J.D. (2015). Cyclic di-GMP modulates gene expression in Lyme disease spirochetes at the tick-mammal interface to promote spirochete survival during the blood meal and tick-to-mammal transmission. *Infect Immun* 83**,** 3043-3060.

Caimano, M.J., Iyer, R., Eggers, C.H., Gonzalez, C., Morton, E.A., Gilbert, M.A., Schwartz, I., and Radolf, J.D. (2007). Analysis of the RpoS regulon in *Borrelia burgdorferi* in response to mammalian host signals provides insight into RpoS function during the enzootic cycle. *Mol Microbiol* 65**,** 1193-1217.

Caimano, M.J., Yang, X., Popova, T.G., Clawson, M.L., Akins, D.R., Norgard, M.V., and Radolf, J.D. (2000). Molecular and evolutionary characterization of the cp32/18 family of supercoiled plasmids in *Borrelia burgdorferi* 297. *Infection and Immunity* 68**,** 1574-1586.

Casjens, S., Van Vugt, R., Tilly, K., Rosa, P.A., and Stevenson, B. (1997). Homology throughout the multiple 32-kilobase circular plasmids present in Lyme disease spirochetes. *Journal of Bacteriology* 179**,** 217-227.

Casjens, S.R., Mongodin, E.F., Qiu, W.G., Luft, B.J., Schutzer, S.E., Gilcrease, E.B., Huang, W.M., Vujadinovic, M., Aron, J.K., Vargas, L.C., Freeman, S., Radune, D., Weidman, J.F., Dimitrov, G.I., Khouri, H.M., Sosa, J.E., Halpin, R.A., Dunn, J.J., and Fraser, C.M. (2012). Genome stability of Lyme disease spirochetes: comparative genomics of *Borrelia burgdorferi* plasmids. *PLoS One* 7**,** e33280.

Disz, T., Akhter, S., Cuevas, D., Olson, R., Overbeek, R., Vonstein, V., Stevens, R., and Edwards, R.A. (2010). Accessing the SEED genome databases via Web services API: tools for programmers. *BMC Bioinformatics* 11**,** 319.

Eggers, C.H., Caimano, M.J., and Radolf, J.D. (2004). Analysis of promoter elements involved in the transcriptional initiation of RpoS-dependent *Borrelia burgdorferi* genes. *J Bacteriol* 186**,** 7390-7402.

Eggers, C.H., Caimano, M.J., and Radolf, J.D. (2006). Sigma factor selectivity in *Borrelia burgdorferi*: RpoS recognition of the *ospE*/*ospF*/*elp* promoters is dependent on the sequence of the -10 region. *Mol Microbiol* 59**,** 1859-1875.

Iyer, R., Caimano, M.J., Luthra, A., Axline, D., Jr., Corona, A., Iacobas, D.A., Radolf, J.D., and Schwartz, I. (2015). Stage-specific global alterations in the transcriptomes of Lyme disease spirochetes during tick feeding and following mammalian host adaptation. *Mol Microbiol* 95**,** 509-538.

Letunic, I., and Bork, P. (2016). Interactive tree of life (iTOL) v3: an online tool for the display and annotation of phylogenetic and other trees. *Nucleic Acids Res* 44**,** W242-245.

Lin, T., Gao, L., Zhang, C., Odeh, E., Jacobs, M.B., Coutte, L., Chaconas, G., Philipp, M.T., and Norris, S.J. (2012). Analysis of an ordered, comprehensive STM mutant library in infectious *Borrelia burgdorferi*: insights into the genes required for mouse infectivity. *PLoS One* 7**,** e47532.

Ouyang, Z., Blevins, J.S., and Norgard, M.V. (2008). Transcriptional interplay among the regulators Rrp2, RpoN and RpoS in *Borrelia burgdorferi*. *Microbiology* 154**,** 2641-2658.

Ouyang, Z., Deka, R.K., and Norgard, M.V. (2011). BosR (BB0647) controls the RpoN-RpoS regulatory pathway and virulence expression in *Borrelia burgdorferi* by a novel DNA-binding mechanism. *PLoS Pathog* 7**,** e1001272.

Sievers, F., Wilm, A., Dineen, D., Gibson, T.J., Karplus, K., Li, W., Lopez, R., Mcwilliam, H., Remmert, M., Soding, J., Thompson, J.D., and Higgins, D.G. (2011). Fast, scalable generation of high-quality protein multiple sequence alignments using Clustal Omega. *Mol Syst Biol* 7**,** 539.

Yang, X., Popova, T.G., Hagman, K.E., Wikel, S.K., Schoeler, G.B., Caimano, M.J., Radolf, J.D., and Norgard, M.V. (1999). Identification, characterization, and expression of three new members of the *Borrelia burgdorferi* Mlp (2.9) lipoprotein gene family. *Infect Immun* 67**,** 6008-6018.

Zuckert, W.R., Lloyd, J.E., Stewart, P.E., Rosa, P.A., and Barbour, A.G. (2004). Cross-species surface display of functional spirochetal lipoproteins by recombinant *Borrelia burgdorferi Infection and Immunity* 72**,** 1463-1469.
